# Triphenylamine-Based Supramolecular Coordination Metallacycles

**DOI:** 10.3390/molecules31152652

**Published:** 2026-07-30

**Authors:** Keyu Ai, Zichen Bu, Yi-Xiong Hu, Sai Li

**Affiliations:** 1 School of Safety Science and Engineering, Xi’an University of Science and Technology, 58 N. Yanta Middle Road, Xi’an 710054, China; 21120089006@stu.xust.edu.cn; 2 Shaanxi Key Laboratory of Low Metamorphic Coal Clean Utilization, School of New Energy, Yulin University, 51 N. Chongwen Road, Yulin 719000, China; buzichen@stu.yulinu.edu.cn

**Keywords:** coordination-driven self-assembly, metallacycle, triphenylamine, tunable fluorescence

## Abstract

Over the past two decades, triphenylamine (TPA)-based supramolecular coordination metallacycles, constructed by incorporating TPA and its derivatives as key building blocks into two-dimensional (2D) coordination-driven assemblies, have gradually received considerable attention and emerged as an important research topic within the field of discrete supramolecular coordination complexes (SCCs). Leveraging the synthetic accessibility and outstanding optoelectronic properties of TPA units, the resulting TPA-based metallacycles exhibit well-defined topological structures, excellent emission characteristics, and unique functions. In this review, we systematically and comprehensively discuss the design strategies, synthetic methodologies, and practical applications for TPA-based supramolecular metallacycles, with an emphasis on five key aspects: (i) coordination-driven self-assembly, (ii) hierarchical self-assembly, (iii) supramolecular polymers, (iv) tunable fluorescence, and (v) diverse applications. Finally, future perspectives and challenges in this rapidly evolving field are presented.

## 1. Introduction

Since the mid-1990s, coordination-driven self-assembly, as a simple and effective strategy for accessing discrete supramolecular coordination complexes (SCCs) including two-dimensional (2D) metallacycles and three-dimensional (3D) metallacages, has continued to spark great interest [1,2,3,4,5]. In general, taking advantage of the dynamically reversible nature of metal–ligand bonds which allows self-correction during assembly processes, the resultant SCCs possess well-defined topological architectures (sizes, shapes and geometries) and are obtained in nearly quantitative yields [6,7,8,9,10]. To date, numerous intriguing SCCs with novel geometrical structures have been produced using such a strategy. These developed SCCs have exhibited a wide range of functions across multiple research areas, from chemistry to biology, and even materials science [11,12,13,14,15,16,17,18].

As one of the most important SCCs, 2D metallacycles are characterized by the closed ring within their structure and consist of organic building blocks linked to metal ions or metal-containing organic ligands via metal–ligand bonds [18,19,20,21]. In sharp contrast to traditional macrocycles such as crown ethers, cyclodextrin, and cucurbituril, metallacycles can not only serve as hosts to encapsulate guests, but also enable the convenient preparation of functionalized self-assembly systems by introducing functional building blocks into coordination precursors. During the past few decades, supramolecular chemists have been devoted to creating a variety of metallacycles, some of which have shown a range of applications including supramolecular catalysis [22,23,24,25,26,27,28,29], optical materials [30,31,32,33,34,35,36,37,38], polymers [39,40,41,42,43,44], liquid crystals [45,46,47,48], tumor treatment [49,50,51,52,53,54,55], etc. [56,57,58,59,60,61]. It should be noted that the skeletons of metallacycles are relatively planar, rigid, and often positively charged, which is especially helpful for designing and constructing highly ordered self-assembly structures, making them excellent candidates for the fabrication of hierarchical self-assembly systems through combination with other noncovalent interactions [62,63,64]. More importantly, an attractive aspect particularly worth exploring is the difference in properties between functional assembly precursors and the formed metallacycles arising from coordination interactions as well as structural effects.

Recently, the introduction of TPA units into 2D supramolecular coordination metallacycles, giving rise to so-called TPA-based metallacycles, has received widespread interest and gradually become one of the most popular research focuses in the field of SCCs. The synthetic accessibility, structural stability and flexibility of TPA derivatives are crucial factors in preparing TPA-based metallacycles [65,66,67,68,69]. On one hand, TPA moieties, with their relatively rigid structure and an approximate 120° angle between every two phenyl groups, are dramatically suitable to constitute the core scaffolds of the targeted metallacycles. On the other hand, they can act as key building blocks attached to the skeletons of the macrocyclic outcomes. In addition, the investigation of these TPA-based metallacycles for practical applications is another attractive aspect. The nonplanar conjugated structure with electron-rich features of TPA and its derivatives has aroused great interest in exploring the optoelectronic properties of the resulting TPA-based metallacycles [70,71,72,73]. Meanwhile, utilizing the photoelectric response behavior of TPA units embedded in metallacycles to construct stimuli-responsive functional systems, such as metallacycle-containing polymers and hierarchical self-assembly materials, is extremely significant.

To date, a systematic overview focusing specifically on TPA-based supramolecular metallacycles is still lacking. A large number of comprehensive reviews have summarized the design principles and applications of SCCs in general, while the unique structural features of TPA, including its propeller-shaped geometry, electron-rich nature, and convenient structural modification, distinguish this class of metallacycles from other SCCs and necessitate an independent review. Moreover, the explosive progress of this field in recent years, with numerous new examples and emerging applications reported, highlights the urgent need for a timely dedicated collation of relevant advances.

In this review, we highlight the key role of TPA and its derivatives in the straightforward construction of 2D TPA-based supramolecular metallacycles, with the aim of introducing readers to current achievements and future developments. Representative examples are selected to illustrate their design strategies, synthetic methods, optoelectronic properties, and practical applications, covering five key aspects: coordination-driven self-assembly, hierarchical self-assembly, supramolecular polymers, tunable fluorescence, and diverse applications. It should be noted that TPA-based metallacages have also been investigated to some extent [74,75,76,77,78,79,80,81,82,83,84,85,86]. However, they are not discussed in this review. We anticipate that the presented discussions may stimulate the further development of TPA-based metallacycles and SCCs in general.

## 2. Synthesis and Assembly of TPA-Based Metallacycles

The successful construction of discrete supramolecular coordination metallacycles relies on the precise matching between complementary building blocks through coordination bonds [1,12,18]. Building units with well-defined geometric parameters, such as specific angles, symmetry elements, and coordination vectors, are combined in predetermined stoichiometric ratios to afford the target architectures. TPA has proven to be a particularly versatile scaffold in this field [66,73]. By introducing coordination sites at appropriate positions, TPA derivatives can function either as organic donors or as part of metal-containing acceptors. Moreover, the ease of chemical modification of the TPA core allows fine-tuning of the electronic properties and steric profiles of the resulting ligands. This flexibility has enabled the assembly of a wide range of 2D TPA-based metallacycles with tunable sizes, shapes, and functions, as will be discussed in the following sections.

### 2.1. Coordination-Driven Self-Assembly of TPA-Based Metallacycles

**Scheme 1:**
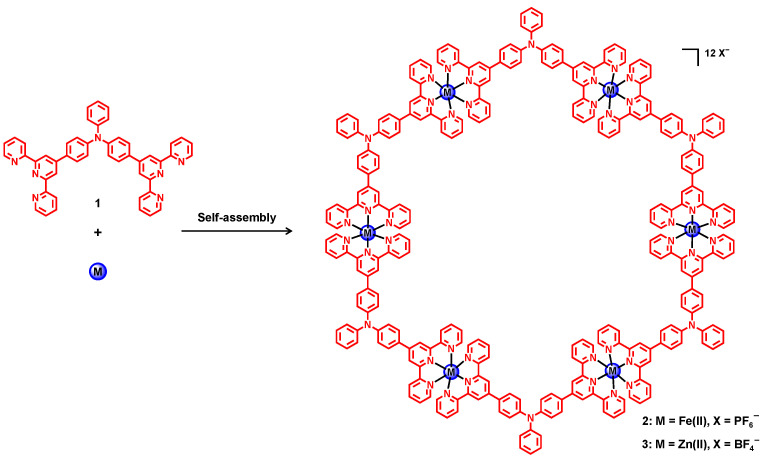
Coordination-driven self-assembly of TPA-based metallacycles **2** and **3**. Reagents and conditions: for metallacycle **2**, (i) FeCl_2_·4H_2_O, MeOH, 12 h, (ii) NH_4_PF_6_/MeOH; for metallacycle **3**, Zn(BF_4_)_2_·8H_2_O, MeCN, 80 °C, 24 h.

2,2′:6′,2″-Terpyridine ligands are widely utilized as fundamental building blocks for the fabrication of supramolecular assemblies and macromolecular systems [9]. On the basis of the coordination-driven self-assembly strategy, Newkome et al. reported the construction of TPA-based metallacycles in 2006 by employing the bis(terpyridine) ligand 4,4′-bis(2,2′:6′,2″-terpyridinyl)triphenylamine **1** (Scheme 1) [87]. Single-crystal X-ray diffraction analysis of ligand **1** revealed that the two terpyridine units adopt an approximately coplanar arrangement with an inter-arm angle of 119.69°, which is well suited for the formation of hexagonal architectures. Through coordination with transition metal ions, well-defined hexameric metallacycles **2** and **3** were successfully obtained. Specifically, metallacycle **2** was readily generated via the self-assembly of ligand **1** with FeCl_2_·4H_2_O in methanol within 12 h. Under similar conditions, treatment of ligand **1** with Zn(BF_4_)_2_·8H_2_O (1:1 ratio) in acetonitrile at 80 °C for 24 h afforded the analogous metallacycle **3** as a yellow semicrystalline solid in 55% yield. The resulting metallacycles were further characterized by NMR, mass spectrometry, and UV–vis spectroscopy, exhibiting distinct photophysical properties. Moreover, photoelectrochemical studies demonstrated that metallacycles **2** and **3** could serve as potential sensitizers for solar cell applications. This result highlights the feasibility and potential of TPA units as key structural motifs for constructing functional supramolecular coordination metallacycles.

**Scheme 2:**
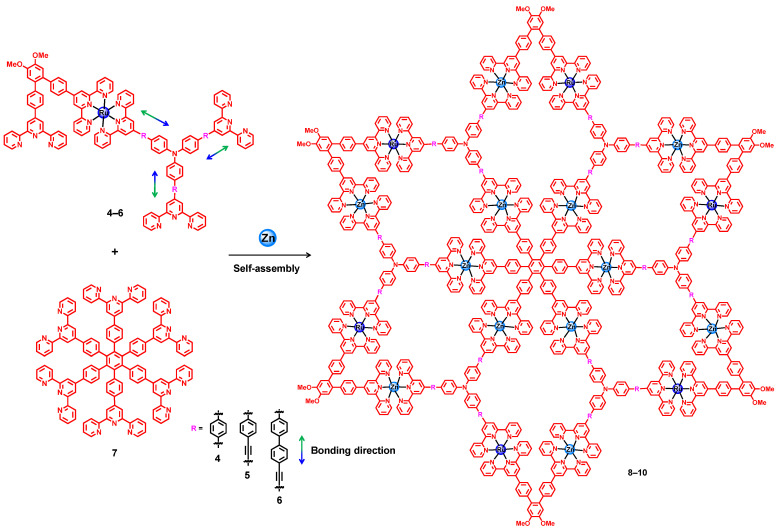
Coordination-driven self-assembly of TPA-based metallacycles **8**–**10**. The structures of ligands **4**–**6** with different arm lengths are presented. Reagents and conditions: (i) CHCl_3_/MeOH, 50 °C, 5 h, (ii) NH_4_PF_6_.

The variation in coordination strength between 2,2′:6′,2″-terpyridine ligands and transition metal ions provide a basis for stepwise assembly, enabling the construction of complex and highly symmetric supramolecular coordination structures [88,89]. For example, Li and co-workers reported the construction of three snowflake-like TPA-based metallacycles **8**–**10** with different sizes via coordination-driven self-assembly (Scheme 2) [90]. Specifically, preorganized Ru(II)-based ligands **4**–**6** were combined with a hexatopic ligand **7** and Zn(NO_3_)_2_·6H_2_O in a stoichiometric ratio of 6:1:12 in a CHCl_3_/MeOH mixture at 50 °C for 5 h. The resulting metallacycles were isolated as red precipitates upon the addition of excess NH_4_PF_6_ and subsequently purified by repeated washing with deionized water. Structural characterization of metallacycles **8**–**10** was comprehensively carried out using 1D, 2D, and DOSY NMR spectroscopy, electrospray ionization mass spectrometry (ESI-MS), traveling wave ion mobility mass spectrometry (TWIM-MS), and gradient tandem mass spectrometry (gMS^2^). Based on simulated structural models, the sizes of the resulting assemblies were estimated to increase progressively from 9.4 nm for **8** to 10.4 nm for **9** and 11.8 nm for **10**.

**Scheme 3:**
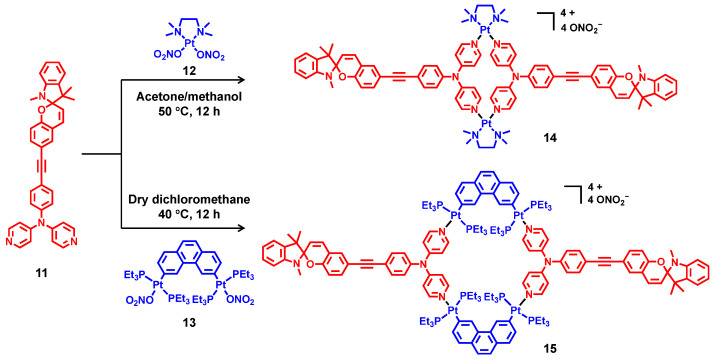
Coordination-driven self-assembly of TPA-based metallacycles **14** and **15**.

In 2020, Stang and Mukherjee et al. reported the construction of two TPA-based metallacycles via coordination-driven self-assembly of dipyridyl donors with organoplatinum(II) acceptors [91]. As shown in Scheme 3, the dipyridyl ligand **11** was treated with cis-protected (tmen)Pt(NO_3_)_2_ (**12**, tmen = N,N,N′,N′-tetramethylethane-1,2-diamine) in an acetone/methanol (1:1, *v*/*v*) mixture at 50 °C for 12 h, affording the corresponding metallacycle **14**. In a parallel approach, ligand **11** was reacted with a 60° diplatinum(II) acceptor **13** in dry dichloromethane at 40 °C for 12 h, and subsequent diffusion of diethyl ether into the concentrated solution yielded the discrete metallacycle **15** as a precipitate. In both cases, the formation of single, well-defined metallacycles was confirmed by multinuclear NMR spectroscopy (^1^H, ^31^P, ^13^C, and 2D DOSY) and ESI-MS. Notably, incorporation of spiropyran units endowed the resulting metallacycles with photoresponsive behavior, enabling reversible structural transformations upon light irradiation and demonstrating efficient photoswitching performance.

**Scheme 4:**
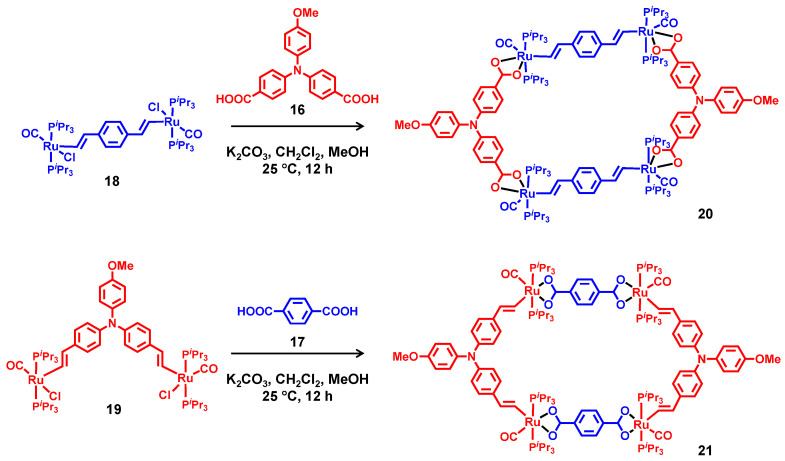
Coordination-driven self-assembly of metallacycles **20** and **21**.

In addition, Winter and co-workers designed and synthesized TPA-based metallacycles **20** and **21** via the coordination-driven self-assembly of spatially extended dicarboxylate ligands **16** and **17** with dinuclear bis(alkenyl) diruthenium precursors **18** and **19** (Scheme 4) [92]. In a typical procedure, the dicarboxylic acid was dissolved in methanol and deprotonated with potassium carbonate (2 equiv.), followed by dropwise addition to a dichloromethane solution of the corresponding diruthenium complex in a 1:1 stoichiometric ratio. The reaction mixtures were stirred for 12–48 h with continuous NMR monitoring until a single discrete product was formed. After standard workup, the desired metallacycles **20** and **21** were isolated as yellow solids in moderate yields (60–70%). The structures of these macrocycles were confirmed by NMR spectroscopy and ultra-high-resolution ESI-MS. Single-crystal X-ray structure determination analysis revealed that metallacycle 20 crystallizes as a hexabenzene solvate in the space group *P*2_1_/*n*, with an oval-shaped inner cavity of approximately 17.0 × 10.0 Å. Their redox properties were further investigated by cyclic and square-wave voltammetry, while spectroelectrochemical studies (IR/NIR and UV–vis/NIR) and electron paramagnetic resonance (EPR) spectroscopy provided insights into their oxidized states.

Collectively, these examples illustrate that TPA-based metallacycles can be accessed through diverse coordination-driven self-assembly strategies, including terpyridine–metal coordination, Pt–N and Pt–O bonds, and Ru–carboxylate coordination. While most reported systems are assembled through a single type of metal–ligand interaction, the combination of orthogonal coordination motifs may provide a viable route toward more sophisticated supramolecular architectures with improved structural stability and functional versatility.

### 2.2. Hierarchical Self-Assembly of TPA-Based Metallacycles

Hierarchical self-assembly refers to the stepwise organization of discrete building blocks into higher-order superstructures driven by secondary noncovalent interactions, a strategy widely employed in nature to create complex functional architectures [62,63,64]. In the context of TPA-based metallacycles, the propeller-shaped TPA core not only imparts excellent optoelectronic properties but also facilitates multiple noncovalent interactions, including π–π stacking, CH–π contacts, hydrophobic effects, and even radical–radical coupling upon oxidation. These interactions enable TPA-containing metallacycles to hierarchically assemble into diverse nanostructures.

**Scheme 5:**
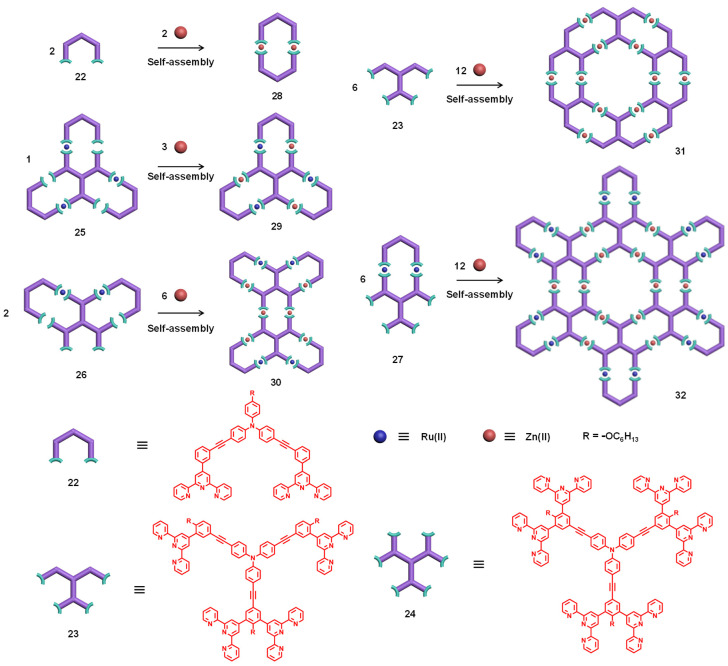
Coordination-driven self-assembly of metallacycles **28**–**32**.

**Figure 1:**
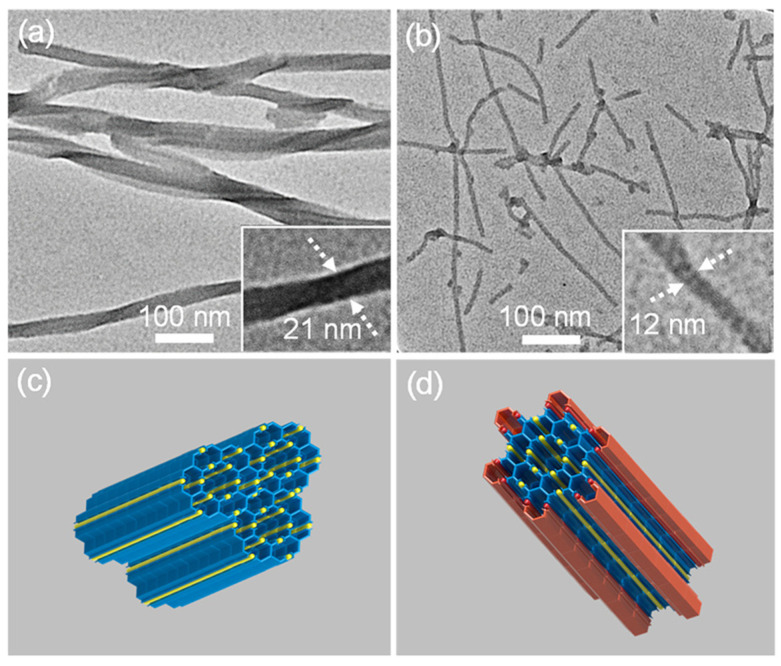
TEM images of hierarchical self-assembly nanostructures formed by (**a**) metallacycle **31** and (**b**) metallacycle **32** in solution and (**c**,**d**) proposed packing models ((**c**) for **31**, (**d**) for **32**). Reproduced with permission [93]. Copyright 2018 American Chemical Society.

In 2018, Li and co-workers reported the construction of five generations of discrete TPA-based metallacycles **28**–**32** based on coordinating organic or Ru(II)-containing building blocks **22**–**27** with Zn(II) ions (Scheme 5) [93]. The TPA motif served as a geometry-controlling core, while terpyridine units provided coordination sites. For example, metallacycle **28** was formed from ligand **22** and Zn(II) ions, while metallacycles **31** and **32** were assembled from larger ligands **23** and **27**, respectively. Similarly, metallacycles **29** and **30** were assembled from Ru(II)-containing building blocks **25** and **26**, respectively, with Zn(II) ions. The assemblies were characterized by 1D/2D NMR, ESI-MS, TWIM-MS, and TEM. Notably, metallacycles **31** and **32** further underwent hierarchical self-assembly. In solution, metallacycle **31** organized into columnar nanostructures that bundled into twisted fiber-like aggregates (Figure 1a), whereas metallacycle **32** formed tubular structures with a diameter of approximately 12 nm (Figure 1b). These morphologies were attributed to multiple noncovalent interactions, including π–π and CH–π stacking, and proposed packing models (Figure 1c,d) suggested that the size and orientation of the metallacyclic scaffolds dictate the distinct assembly behaviors, with the more compact geometry of **31** favoring unidirectional stacking into fibrous aggregates and the larger scaffold of **32** promoting curvature into tubular structures.

**Scheme 6:**
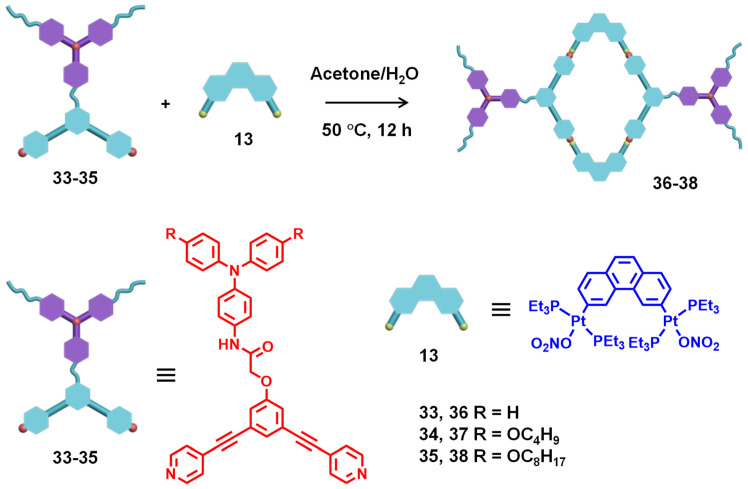
Self-assembly of 120° TPA-based dipyridyl donor ligands **33**–**35** and 60° di-Pt(II) acceptor **13** into hexagonal metallacycles **36**–**38**.

**Figure 2:**
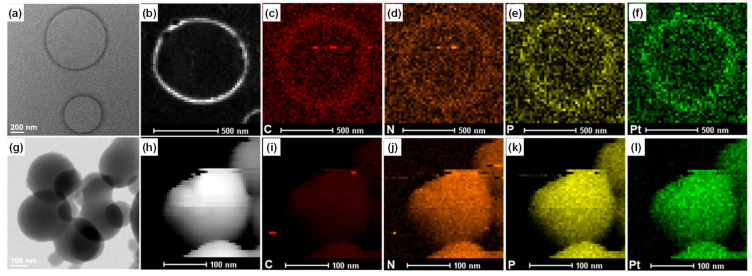
TEM images of hierarchical self-assembly of metallacycle **38** in (**a**) the solution state and (**g**) solid state. HAADF STEM image of hierarchical self-assembly of metallacycle **38** in (**b**) the solution state and (**h**) solid state. The elemental mappings of hierarchical self-assembly of metallacycle **38** in (**c**–**f**) the solution state and (**i**–**l**) solid state. Reproduced with permission [94]. Copyright 2019 American Chemical Society.

The reversible redox property of TPA units offers an additional avenue to induce hierarchical self-assembly of TPA-containing metallacycles. In 2019, Yang et al. constructed three rhomboidal metallacycles **36**–**38** via the coordination of a 120° dipyridyl donor bearing TPA units **33**–**35** with a 60° diplatinum(II) acceptor **13**, respectively (Scheme 6) [94]. The TPA moieties were functionalized with different alkyl chains. Metallacycle **36** had no alkyl chain, **37** carried medium-length chains, and **38** featured long alkyl chains. Upon white-light irradiation in chloroform, UV-vis, EPR, and NMR measurements showed that metallacycle **38** generated stable TPA radical cations, whereas metallacycle **36** produced no radicals and metallacycle **37** generated radicals that rapidly degraded. As a result, metallacycle **38** underwent radical-induced hierarchical self-assembly. In solution, TEM and HAADF-STEM imaging of metallacycle **38** revealed vesicular structures with a hollow core, confirmed by elemental mapping of carbon, nitrogen, phosphorus, and platinum within the ring-shaped assemblies (Figure 2a–f). DLS measurements of metallacycle **38** in solution further confirmed the assembly process, showing a significant increase in hydrodynamic diameter from 1.5 nm to 332.5 nm upon irradiation. In the solid state, TEM, HAADF-STEM images and elemental mapping of metallacycle **38** showed sphere micelles, distinct from the vesicles observed in solution, which was attributed to enhanced π–π stacking interactions in the absence of solvent (Figure 2g–l). Coarse-grained molecular dynamics simulations indicated that the assembly was driven by a combination of TPA radical–radical interactions and π–π stacking between metallacycle backbones. The process was reversible upon heating, and the long alkyl chains were essential for stabilizing the radicals and enabling assembly.

**Figure 3:**
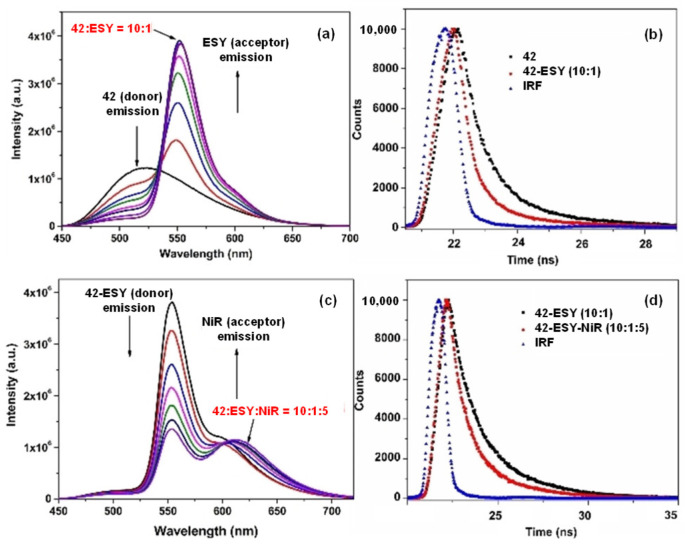
(**a**) Fluorescence spectra of **42** with gradual addition of **ESY** (*λ*_ex_ = 410 nm). (**b**) Change in the fluorescence decay profiles of **42** in presence of **ESY** (**42**:**ESY** = 10:1). (**c**) Fluorescence spectra with gradual addition of **NiR** (*λ*_ex_ = 410 nm) and (**d**) fluorescence lifetime decay profile in presence of **NiR** of **42-ESY** assembly (**42:ESY:NiR** = 10:1:5). All measurements were performed in water-acetone (9:1, *v*/*v*). Reproduced with permission [96]. Copyright 2022 John Wiley and Sons.

**Scheme 7:**
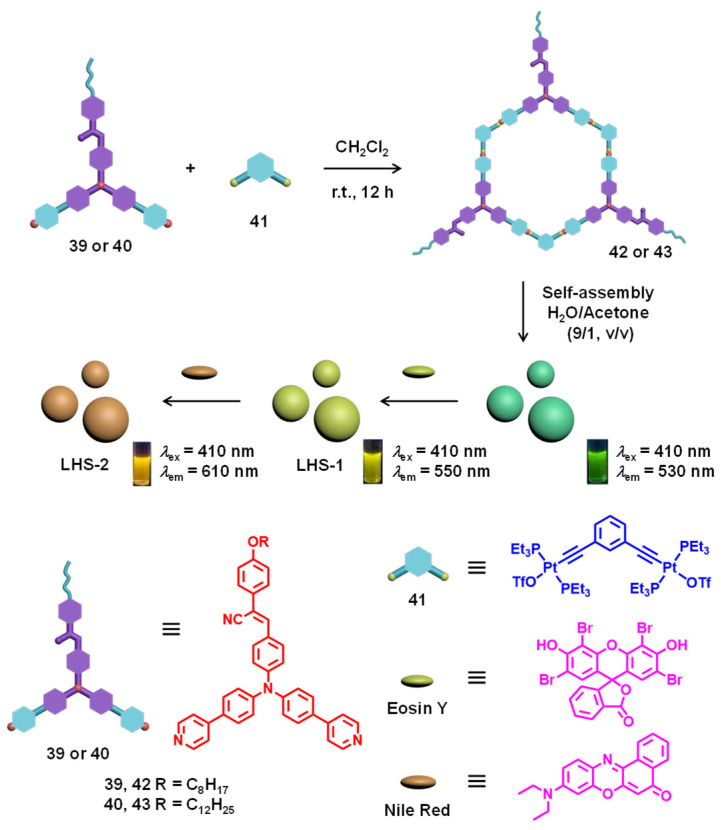
A cartoon representation of the construction of metallacycle-based LHSs with two-step sequential energy transfer based on hierarchical self-assembly strategy.

Recently, Acharyya, Mukherjee, Stang and co-workers developed a series of fluorescent Pt(II) metallacycles based on TPA and cyanostilbene-cored ligands [95,96]. These metallacycles underwent hierarchical self-assembly into nanospheres in mixed solvents with aggregation-induced emission enhancement (AIEE) and served as a platform to encapsulate hydrophobic dye molecules for constructing artificial light-harvesting systems (LHSs) via Förster resonance energy transfer (FRET). For example, ligand **39** bearing a TPA-based cyanostilbene moiety with a shorter alkyl chain was coordinated with the 180° di-Pt(II) acceptor **41** to afford hexagonal metallacycle **42**, while ligand **40** bearing a longer alkyl chain similarly produced metallacycle **43** (Scheme 7) [96]. These metallacycles hierarchically assembled into nanospheres in water–acetone mixtures, as confirmed by TEM and SEM analyses. DLS measurements revealed average hydrodynamic diameters of approximately 228 nm for **42** and 177 nm for **43**, indicating that the longer alkyl chain of **43** promotes more compact aggregate formation. The AIEE characteristics observed for the aggregates of these metallacycles arise from the TPA–cyanostilbene conjugated system, where restricted intramolecular rotation in the aggregated state suppresses nonradiative decay pathways. Notably, the longer alkyl chain of metallacycle **43** promotes tighter molecular packing, resulting in more compact nanospheres with a higher degree of AIEE enhancement relative to its shorter-chain counterpart metallacycle **42**. Figure 3a shows that upon gradual addition of Eosin Y (**ESY**) to the hierarchical assembly solution, the emission intensity of metallacycle **42** decreased while that of **ESY** increased, indicating efficient energy transfer from donor metallacycle **42** to acceptor **ESY**. The energy transfer efficiency reached 80.1% for **42** and 76.4% for **43**, and the fluorescence lifetime of metallacycle **42** was significantly shortened in the presence of **ESY** (Figure 3b). When a second dye, Nile Red (**NiR**), was added, a two-step cascade energy transfer from metallacycle **42** to **ESY** and then to **NiR** occurred. This was evidenced by the decrease in **ESY** emission and the appearance of **NiR** emission (Figure 3c), along with a further lifetime reduction (Figure 3d). In this relay mode, **ESY** acted as a bridge. The sequential transfer efficiency was dependent on the alkyl chain length. Metallacycle **42** with shorter chains exhibited a higher efficiency (64.6%) compared to its longer-chain analogue (**43**, 29.7%).

Notably, the hierarchical assembly behavior of TPA-based metallacycles is highly dependent on the nature of peripheral substituents, solvent conditions, and external stimuli. Among the reported examples, the radical-induced assembly of metallacycle **38** represents a unique case where reversible redox chemistry enables dynamic control over nanostructure formation, whereas most other systems rely on conventional noncovalent interactions such as π–π stacking and hydrophobic effects.

## 3. Properties and Applications of TPA-Based Metallacycles

### 3.1. Supramolecular Polymers of TPA-Based Metallacycles

Supramolecular polymers constructed from discrete metallacycles have attracted growing interest due to their combination of well-defined cyclic scaffolds with dynamic, stimuli-responsive properties [39,40,41]. The electrochemical activity and facile chemical modifiability of TPA and its derivatives can be exploited to construct supramolecular polymer systems [65,66,67]. The redox-active nature of TPA enables oxidative electropolymerization, allowing discrete metallacycles to be assembled into stable polymeric films or porous membranes under controlled electrochemical conditions. In addition, the ease of introducing various functional groups or polymerizable handles onto the TPA core facilitates the integration of metallacycles into covalently or dynamically linked polymer networks.

**Scheme 8:**
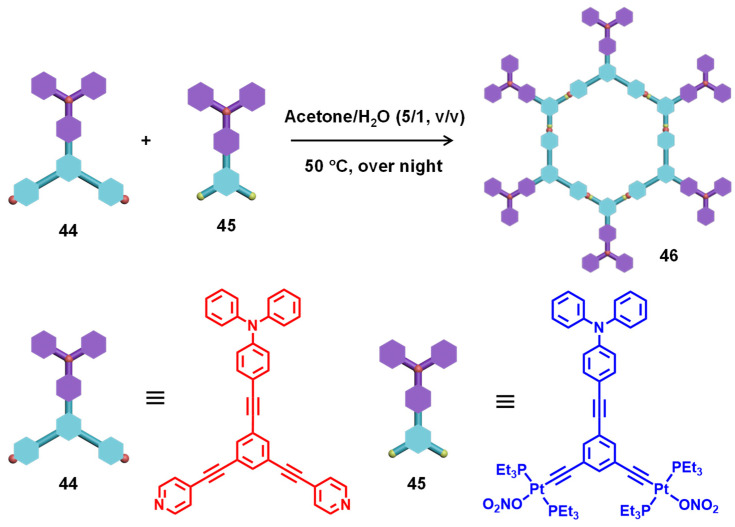
Coordination-driven self-assembly of 120° TPA-based dipyridyl donor ligand **44** and 120° di-Pt(II) acceptor **45** into hexagonal metallacycles **46**.

**Figure 4:**
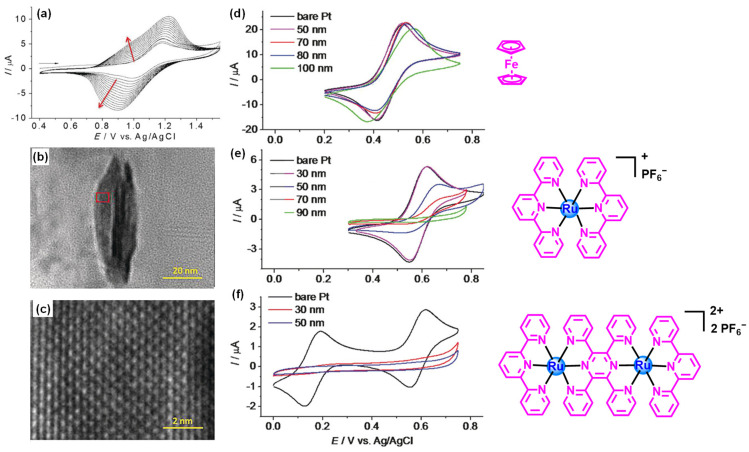
(**a**) Electropolymerization of metallacycle **46** (0.2 mm in CH_2_Cl_2_) at a Pt disc electrode (d = 2 mm, arrows indicate the direction of growth for the peaks as the number of cycles increases). (**b**) TEM micrographs of the thin-film fragment at low magnification and (**c**) the enlarged area. Redox probes used within this study (Right). CV curves of (**d**) ferrocene (2.5 mm), (**e**) medium-sized Ru complex (0.8 mm), and (**f**) bulky Ru dimer (0.5 mm) at a bare Pt disc electrode (d = 2 mm) or a Pt electrode covered with a poly-**46** film with indicated thickness. The measurements were performed in 0.1 M *n*Bu_4_NClO_4_/CH_3_CN solution at a scan rate of 100 mV s^−1^. Reproduced with permission [97]. Copyright 2016 John Wiley and Sons.

In 2016, Yang et al. constructed a hexakis-TPA hexagonal metallacycle **46** by coordination-driven self-assembly of a 120° TPA-functionalized dipyridyl donor **44** and a 120° TPA-containing di-Pt(II) acceptor **45** (Scheme 8) [97]. The structure was confirmed by NMR, CSI-TOF-MS, and PM6 semi-empirical calculations, revealing a well-defined hexagonal cavity with an internal radius of approximately 1.4 nm. Cyclic voltammetry (CV) showed that the six TPA units in the metallacycle underwent oxidative electropolymerization, leading to the deposition of a polymeric film on the electrode surface, as evidenced by the progressive increase in anodic/cathodic peak currents upon repeated potential scans (Figure 4a). The film thickness could be controlled by the number of CV scan cycles, ranging from 30 to 100 nm. TEM revealed a sheet-like morphology with highly ordered lattice planes (Figure 4b,c). The resulting porous membrane exhibited size-selective molecular sieving behavior when tested with redox probes of different molecular sizes. As shown in Figure 4d–f, ferrocene permeated through films up to 100 nm thickness, a medium-sized Ru complex was blocked at 70 nm, and a bulky Ru dimer was fully inhibited even at 30 nm. This work demonstrated that combining coordination-driven self-assembly with electropolymerization of TPA units provides a route to structurally defined porous membranes with tunable thickness and molecular sieving properties.

**Scheme 9:**
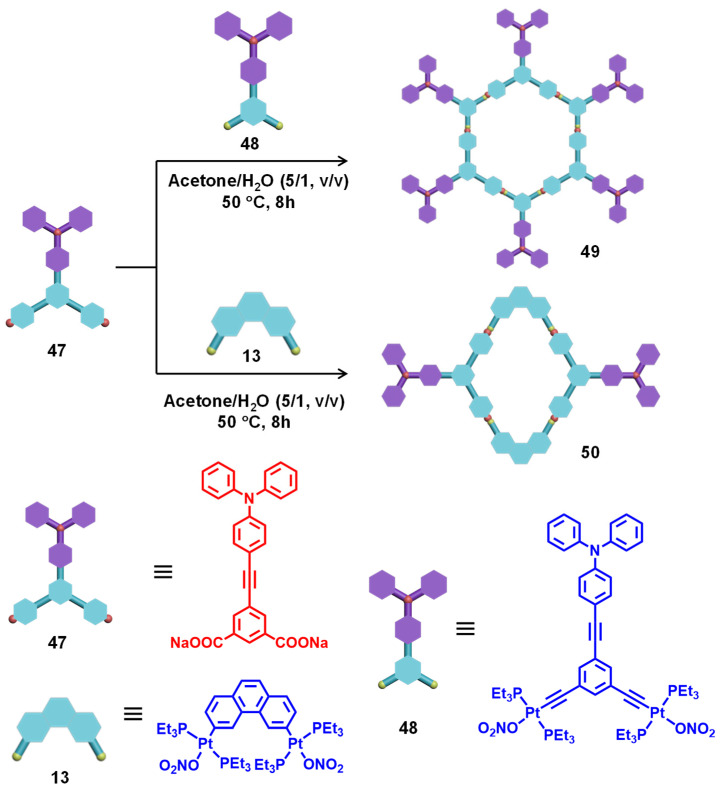
Coordination-driven self-assembly of TPA-based metallacycles **49** and **50**.

**Figure 5:**
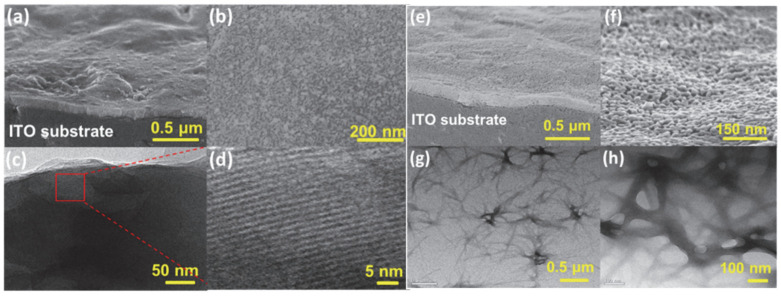
Side view (**a**) and top view (**b**) of representative SEM pictures of poly-**49**/ITO film. TEM micrographs of thin film fragment at low magnification (**c**) and the enlarged area (**d**). Side view (**e**) and top view (**f**) of representative SEM pictures of poly-**50**/ITO film. TEM micrographs of thin-film fragment at low magnification (**g**) and high magnification (**h**). Reproduced with permission [98]. Copyright 2018 John Wiley and Sons.

On the basis of the above work on charged porous membranes, the same group further explored neutral metallacycles and their electropolymerization behavior. In 2018, Yang, Wang and co-workers constructed two neutral multi-TPA metallacycles via coordination-driven self-assembly [98]. The hexagonal metallacycle **49** was obtained from a 120° TPA-substituted dicarboxylate donor **47** and a complementary 120° TPA-functionalized di-Pt(II) acceptor **48**. The rhomboidal metallacycle **50** was assembled from the same dicarboxylate donor **47** and a 60° di-Pt(II) acceptor **13** (Scheme 9). Both metallacycles relied on Pt–O bonds, yielding neutral frameworks without the positive charges typical of pyridine-based analogues. The structures were confirmed by multinuclear NMR spectroscopy, and rhomboid **50** was further characterized by single-crystal X-ray structure determination. CV of **49** and **50** showed that the TPA units underwent oxidative electropolymerization, depositing polymeric films on electrode surfaces. The film thickness could be controlled by the number of scan cycles, ranging from 15 to 70 nm for poly-**49**. Morphological analysis by SEM and TEM revealed that poly-**49** formed smooth, compact sheet-like films, whereas poly-**50** produced fibrous structures (Figure 5). This difference was attributed to the distinct shapes and TPA orientations of the two metallacycle precursors. The resulting neutral polymeric films, free from charged defects, were proposed as promising platforms for the detection, separation, or capture of neutral molecules.

**Scheme 10:**
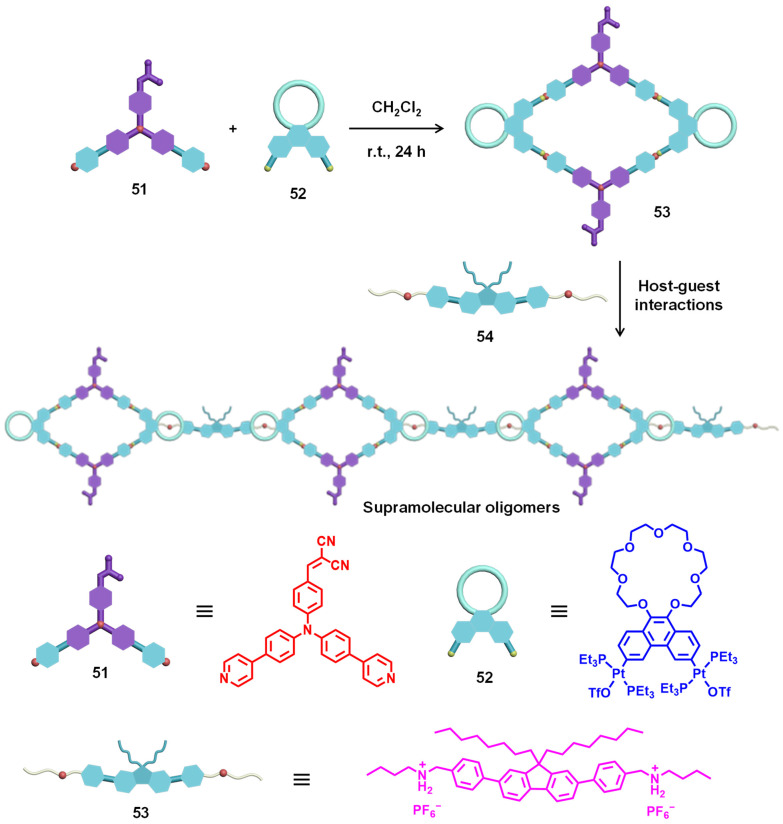
Self-assembly of TPA-based metallacycles **53** and its supramolecular oligomers. The structures of ligands **51**, **52** and **54** are presented.

**Figure 6:**
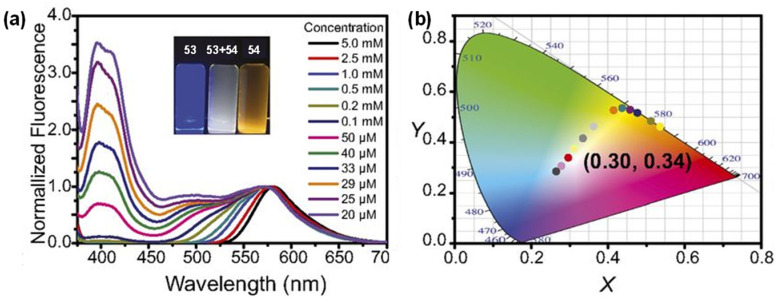
(**a**) Emission spectra of equal molar **53** and **54** at different concentrations; (Inset) photograph of **53**, **54**, and mixture of equal molar **53** and **54** in acetone upon excitation at 365 nm using a UV lamp at 298 K (c = 29 μM). (**b**) CIE chromaticity coordinates of equal molar **53** and **54** at different concentrations, according to the fluorescence spectra recorded in (**a**). Reproduced with permission [99]. Copyright 2019 National Academy of Sciences (USA).

As shown in Scheme 10, Yin, Stang and co-workers constructed a rhomboidal Pt(II) metallacycle **53** using a 120° dipyridyl donor **51** bearing a TPA-based fluorophore and a 60° phenanthrene-21-crown-7 (P21C7)-functionalized di-Pt(II) acceptor **52** [99]. The metallacycle **53** was characterized by NMR and ESI-TOF-MS and exhibited orange emission at 563 nm. The crown ether moieties on **53** served as host sites for a fluorescent bis-ammonium linker **54** through host–guest interactions, forming supramolecular oligomers in acetone. Linker **54** displayed blue emission at 391 and 411 nm. Upon varying the concentration of the 1:1 mixture of **53** and **54**, the emission color changed from orange at high concentration (>0.5 mM) to blue at low concentration (<25 μM) (Figure 6a). This concentration-dependent fluorescence arose from the distinct properties of the two components: metallacycle **53** is AIE active, while linker **54** exhibits aggregation-caused quenching (ACQ). At high concentration, the AIE-dominated emission of **53** showed an orange color; at low concentration, the ACQ linker **54** contributed blue emission. At an intermediate concentration of 29 μM, the sample emitted white light with CIE coordinates of (0.30, 0.34) (Figure 6b). This work provided a strategy to achieve white-light emission by integrating complementary AIE and ACQ fluorophores through orthogonal host–guest interactions.

**Scheme 11:**
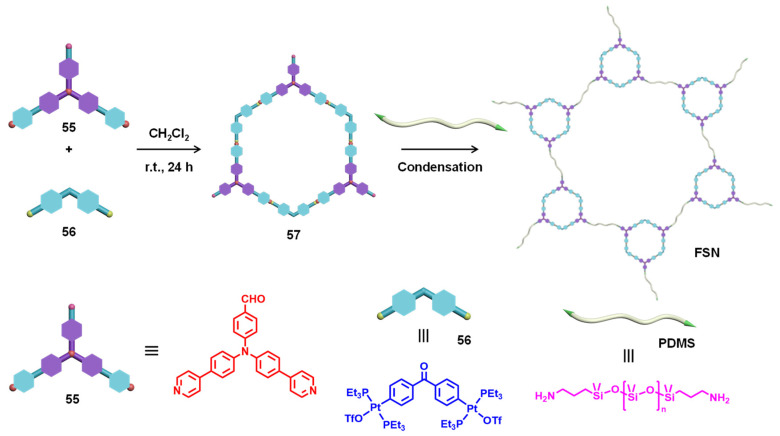
Self-assembly of TPA-based metallacycles **57** and synthetic route of the corresponding fluorescent supramolecular network **FSN**. The structures of ligands **55**, **56** and **PDMS** are presented.

**Figure 7:**
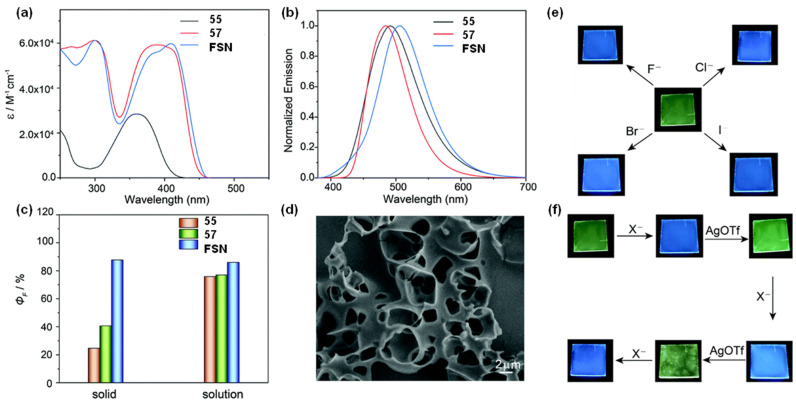
(**a**) UV/vis absorption and (**b**) normalized emission spectra of ligand **55**, metallacycle **57**, and **FSN** in CH_2_Cl_2_ (*λ*_ex_ = 365 nm, c = 10.0 μM). (**c**) Quantum yields of **55**, **57** and **FSN** in solution and in the solid state. (**d**) SEM image of **FSN**. (**e**) Fluorescent photographs of a thin film made by **FSN** before and after the addition of F^−^, Cl^−^, Br^−^ or I^−^. (**f**) Fluorescent photographs of the reversible emission transition of **FSN** (X = F, Cl, Br or I). Reproduced with permission [100]. Copyright 2020 Royal Society of Chemistry.

Dynamic covalent imine bonds offer both stability and reversibility, making them attractive for constructing adaptive supramolecular networks. In 2020, Zhang and co-workers prepared a hexagonal Pt(II) metallacycle **57** by coordinating an aldehyde-functionalized TPA-based dipyridyl ligand **55** with a 60° di-Pt(II) acceptor **56** (Scheme 11) [100]. The structure was confirmed by NMR and ESI-TOF-MS. Subsequently, the six aldehyde groups on metallacycle **57** were condensed with an amine-terminated polydimethylsiloxane (**PDMS**, *M*n = 2000, PDI = 1.18) via dynamic imine bonds, yielding a fluorescent supramolecular network (**FSN**). As shown in Figure 7, the network **FSN** exhibited green emission with a markedly higher quantum yield than the free ligand **55** or the metallacycle **57**, which was attributed to the restricted intramolecular motion of the TPA units upon crosslinking. SEM micrographs revealed an extended porous morphology (Figure 7d). The Pt–N coordination bonds within the network are dynamic; their reversible dissociation upon exposure to halogen ions triggered a rapid emission color change from yellow to blue, as visualized in thin films (Figure 7e,f). This reversible response illustrates how integrating dynamic coordination bonds into metallacycle-based networks can generate smart sensory materials.

Overall, these examples demonstrate that TPA-based metallacycles serve as versatile platforms for constructing functional supramolecular polymers through distinct approaches. The redox-active nature of TPA enables oxidative electropolymerization to generate structurally defined porous membranes with controllable thickness and molecular sieving properties. Alternatively, the installation of crown ether or aldehyde functionalities at the metallacycle periphery allows for orthogonal supramolecular or dynamic covalent polymerization, leading to stimuli-responsive emissive materials with tunable fluorescence. This diversity in polymerization strategies highlights the versatility of TPA-based metallacycles as functional building blocks for constructing polymeric materials.

### 3.2. Tunable Fluorescence of TPA-Based Metallacycles

By introducing various substituents and fluorophores into triphenylamine-based coordination building blocks through a pre-modification strategy, followed by coordination-driven self-assembly, TPA-based metallacycles with tunable fluorescence can be constructed. Depending on the molecular design, these systems can exhibit multicolor emission ranging from blue to orange-red, achieve high fluorescence quantum yields in selected cases, and respond to external stimuli including solvent polarity, temperature, and mechanical force and so on. These diverse and tunable photophysical properties make TPA-based metallacycles promising platforms for smart fluorescent materials.

**Scheme 12:**
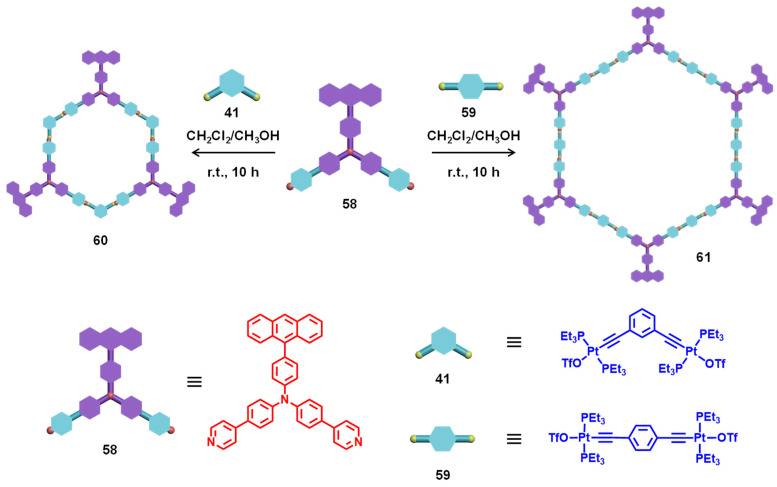
Coordination-driven self-assembly of TPA-based metallacycles **60** and **61**.

**Figure 8:**
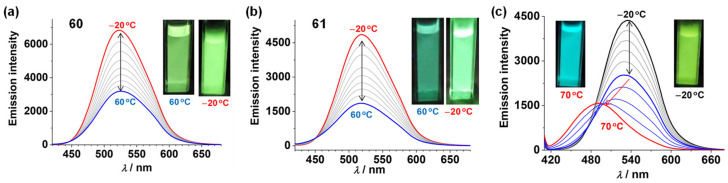
Temperature-responsive emission properties of metallacycle **60** and **61** in THF recorded between −20 and 60 °C. Emission spectral response of (**a**) **60** and (**b**) **61**. The insets show images of the solution under UV illumination (365 nm) at −20 (right) and 60 °C (left). Temperature-responsive emission spectral changes of **60** in DMF recorded between −20 and 70 °C (**c**). The insets show images of the solution under UV illumination (365 nm) at −20 (right) and 70 °C (left). (*λ*_ex_ = 400 nm; c = 2.0 μM; 10 °C temperature intervals) Reproduced with permission [101]. Copyright 2018 American Chemical Society.

In 2018, Stang, Zhong, Saha, and co-workers constructed two hexagonal Pt(II) metallacycles **60** and **61**, via coordination-driven self-assembly of a 120° TPA-based donor **58** bearing an anthracene unit with a 120° or 180° di-Pt(II) acceptor **41** or **59**, respectively (Scheme 12) [101]. Metallacycles **60** and **61** contain three and six freely rotating anthracene pendants, respectively. Their structures were confirmed by multinuclear NMR and ESI-TOF-MS, and PM6 calculations revealed planar hexagonal backbones with cavity sizes of approximately 3.2 nm (**60**) and 6.6 nm (**61**). In THF, the emission intensity of both metallacycles decreased linearly as the temperature increased from −20 to 60 °C, with average sensitivities of −0.67% per °C for **60** and −0.77% per °C for **61** (Figure 8a,b). In contrast, the free ligand **58** showed irregular changes under the same conditions. The reversible response was attributed to temperature-accelerated rotation of the anthracene pendants, which disrupted the charge-transfer absorptions and emissions. In a coordinating solvent such as DMF, a two-step response was observed. Below 30 °C, the behavior was similar to that in THF; above 40 °C, the metallacycles partially decomposed, resulting in a ratiometric emission color change from green to cyan (Figure 8c). This work demonstrated that TPA-based metallacycles can function as reversible fluorescent thermometers with linear temperature sensitivity and, in coordinating solvents, as ratiometric sensors through a self-destruction mechanism.

**Scheme 13:**
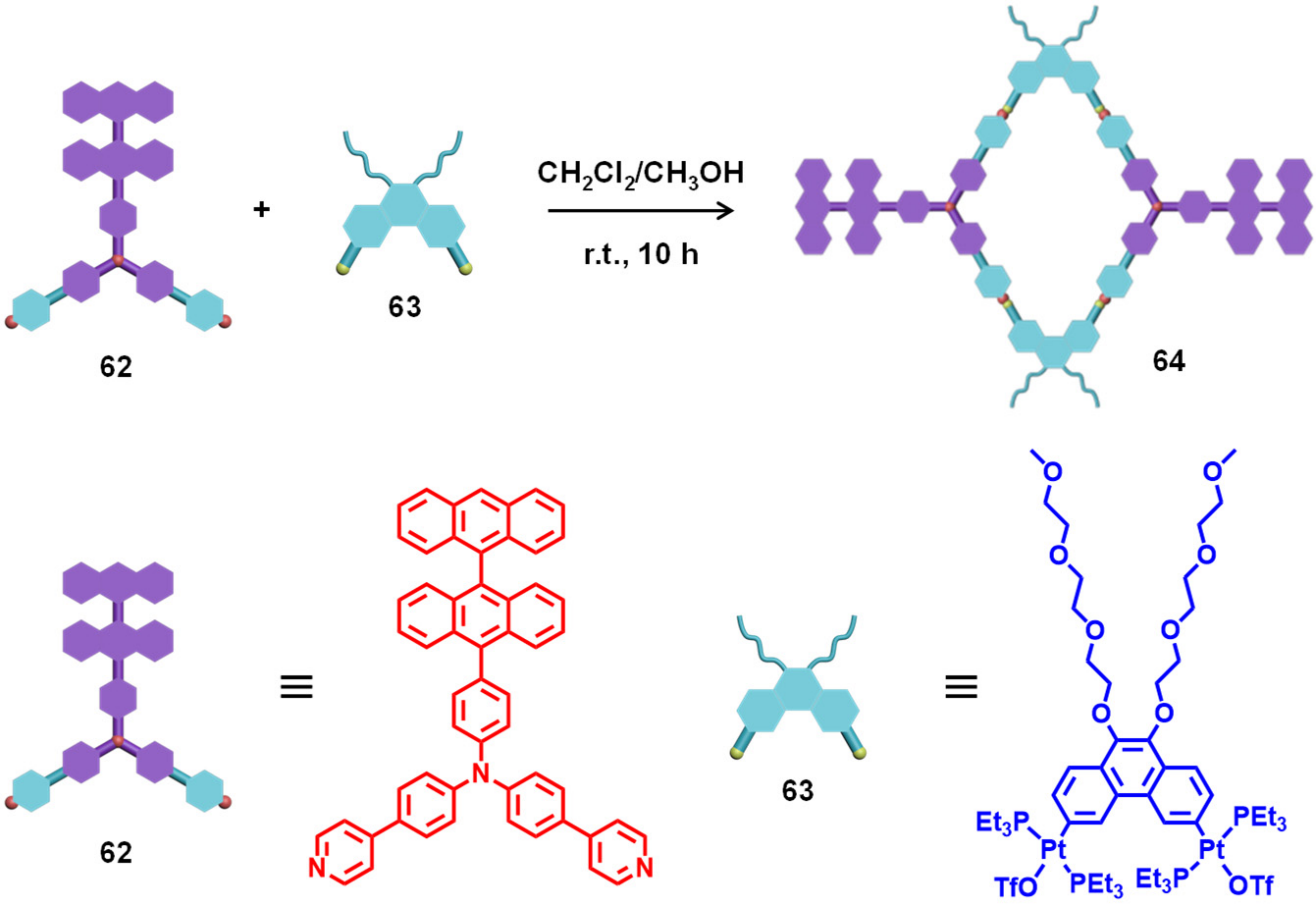
Coordination-driven self-assembly of rhomboidal metallacycles **64**.

**Figure 9:**
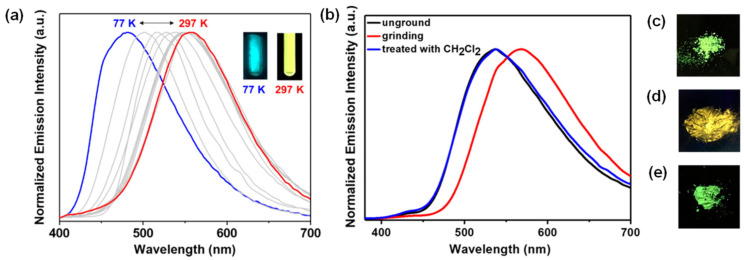
Emission spectra of **64** recorded from 77 to 297 K (20 K intervals) (**a**). Excitation wavelength: 365 nm. The insets show images of the solution under 365 nm irradiation at 77 (left) and 297 K (right). Photoluminescence spectra of **64** before grinding, after grinding, and after treatment with dichloromethane (**b**). Excitation wavelength: 365 nm. Photographs of **64** powder under irradiation by UV light at 365 nm: (**c**) unground **64**; (**d**) ground solid **64**; (**e**) **64** after treatment with dichloromethane solvent vapor. Reproduced with permission [102]. Copyright 2019 American Chemical Society.

Building on the above temperature-responsive metallacycles, Stang, Sun and co-workers further designed a dianthracene-based rhomboidal Pt(II) metallacycle **64** that integrates both temperature and mechanical force responsiveness [102]. As shown in Scheme 13, the 120° dipyridyl donor **62** bearing a bianthracene–triphenylamine moiety was assembled with a 60° di-Pt(II) acceptor **63** to afford the rhomboidal metallacycle **64**. In dilute acetone solution, metallacycle **64** exhibited temperature-dependent fluorescence over a wide range from 77 to 297 K. Specifically, as shown in Figure 9a, the emission maximum gradually red-shifted from 482 nm (cyan) at 77 K to about 540 nm (yellow) at 297 K. In the solid state, the metallacycle displayed reversible mechanochromic behavior (Figure 9b–e). The collected data demonstrate that upon grinding, the emission maximum shifted from 537 nm (green) to 569 nm (yellow), accompanied by a significant drop in quantum yield from 19.3% to 2.8%. The original green emission could be fully restored by exposure to dichloromethane vapor. Powder X-ray diffraction indicated a crystalline-to-amorphous transition upon grinding and full recovery after solvent treatment.

**Scheme 14:**
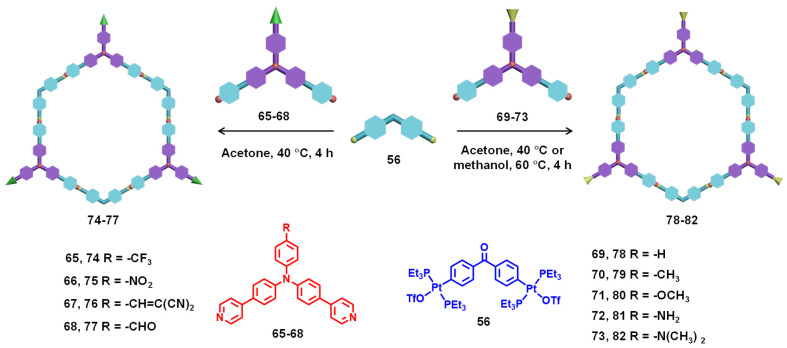
Coordination-driven self-assembly of hexagonal metallacycles **74**–**82**.

**Figure 10:**
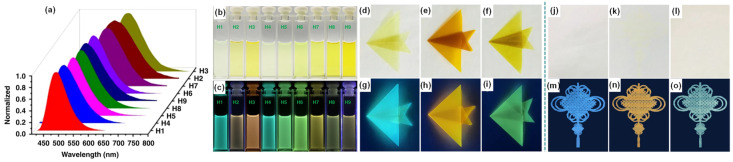
Fluorescence emission spectra of metallacycles **H1**–**H9** in dichloromethane (**a**). Photographs of metallacycles **H1**–**H9** in dichloromethane in visible light (**b**) and under 365 nm light excitation (**c**). Photographs of the flexible fluorescent films (**d**–**i**) and the fluorescent patterns (**j**–**o**) by inkjet printing from metallacycle **H1** in visible light (**d**,**j**) and under 365 nm light excitation (**g**,**m**), metallacycle **H3** in visible light (**e**,**k**) and under 365 nm light excitation (**h**,**n**), metallacycle **H7** in visible light (**f**,**l**) and under 365 nm light excitation (**i**,**o**). Metallacycles **74**–**82** correspond to **H1**–**H9**, respectively. Reproduced with permission [103]. Copyright 2019 Nature Publishing Group.

Introducing different substituents on the TPA core provides an effective way to regulate the photoinduced electron transfer (PET) and intramolecular charge transfer (ICT) properties of the corresponding metallacycles. In 2019, Yang, Xu and co-workers synthesized nine 120° dipyridyl donors **65**–**73** bearing substituents with electron-withdrawing or electron-donating strength (such as –CF_3_, –NO_2_, –CHO, –H, –CH_3_, –OCH_3_, –NH_2_, –N(CH_3_)_2_, and –CH=C(CN)_2_) and assembled them with a 120° di-Pt(II) acceptor **56** to give a series of hexagonal metallacycles **74**–**82** (Scheme 14) [103]. In dichloromethane, the emission wavelengths of **74**–**82** ranged from 480 nm to 590 nm, covering blue, green and orange colors (Figure 10a–c). Metallacycles with electron-withdrawing groups (**74**, **76**, **77**) exhibited enhanced fluorescence quantum yields (up to 61%) compared to their free ligands, because coordination of pyridine to Pt(II) blocked the PET process and avoided the heavy-atom effect. In contrast, those bearing electron-donating groups (**78**–**82**) showed lower quantum yields due to Pt-enhanced intersystem crossing. Benefiting from the tunable emission, metallacycles **74**, **76** and **80** were fabricated into multicolor fluorescent polymer films (Figure 10d–i) and inks for inkjet printing (Figure 10j–o), demonstrating potential in anti-counterfeiting and information storage.

**Scheme 15:**
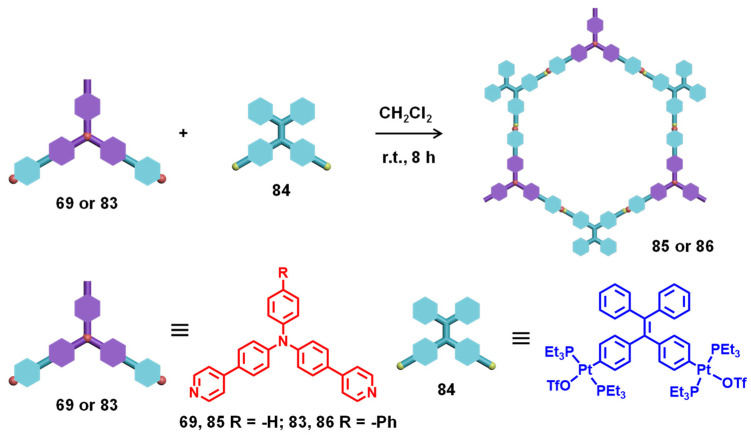
Coordination-driven self-assembly of hexagonal metallacycles **85** and **86**.

**Figure 11:**
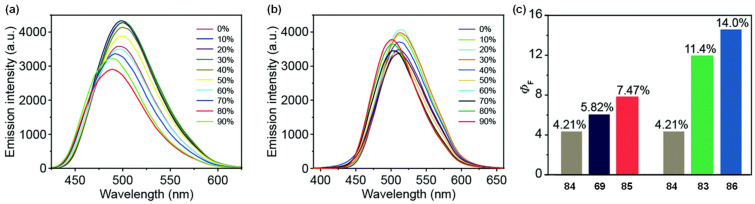
Fluorescence emission spectra of metallacycles **85** (**a**) and **86** (**b**) versus the hexane fraction in CH_2_Cl_2_/hexane mixtures (*λ*_ex_ = 360 nm, c = 10.0 mM). Quantum yields (**c**) of the building blocks (**69** and **83**) and assemblies (**85** and **86**) in the solid state (*λ*_ex_ = 360 nm). Reproduced with permission [104]. Copyright 2021 Royal Society of Chemistry.

In 2021, Yan and co-workers reported a strategy to integrate ACQ and AIE motifs into a single metallacycle, aiming to achieve enhanced fluorescence over a broad concentration range [104]. They assembled two hexagonal Pt(II) metallacycles **85** and **86** by combining a 120° TPE-based di-Pt(II) acceptor **84** with two different 120° TPA-based dipyridyl donors **69** and **83**, respectively (Scheme 15). The structures exhibited planar hexagonal architectures with alternately arranged TPA and TPE units. In dilute CH_2_Cl_2_ solution, both metallacycles showed a red-shifted emission (ca. 60 nm) and lower quantum yields compared to their TPA ligands due to the heavy-atom effect of platinum. The fluorescence behavior of the two metallacycles in CH_2_Cl_2_/hexane mixtures was investigated. As shown in Figure 11a (for **85**) and Figure 11b (for **86**), both complexes exhibited a non-monotonic change. An initial increase in emission intensity accompanied by a red shift occurred at low hexane content, which was attributed to AIE of the TPE units. This was followed by a decrease due to TPA stacking, and then a second enhancement with a blue shift at high hexane fraction, resulting from suppressed charge transfer. Notably, as shown in Figure 11c, the solid-state quantum yields of **85** (7.5%) and **86** (14.0%) were higher than those of their corresponding TPA ligands (5.8% and 11.4%, respectively), demonstrating a synergistic effect between the ACQ and AIE components within the same supramolecular scaffold.

**Scheme 16:**
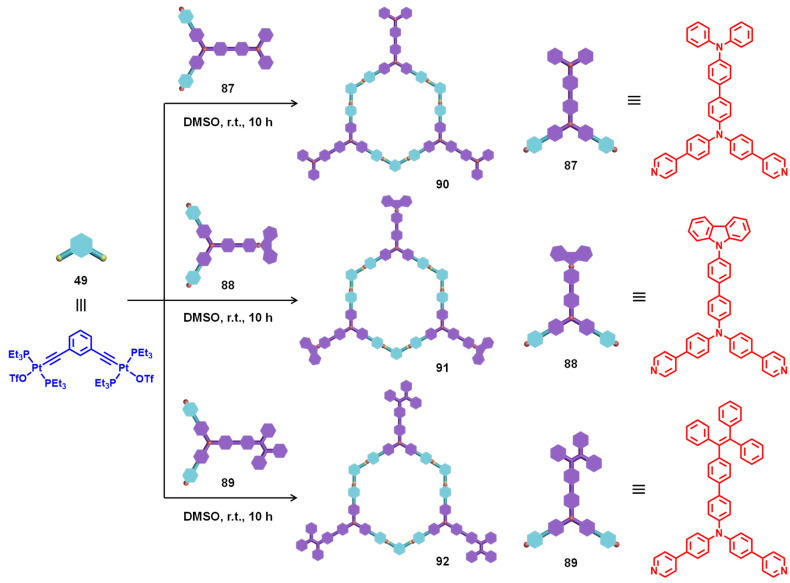
Coordination-driven self-assembly of hexagonal metallacycles **90**–**92**.

**Figure 12:**
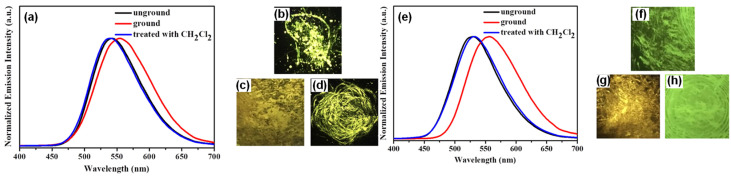
Emission spectra of solid-state metallacycles **91** (**a**) and **92** (**e**) in various states. The excitation wavelength was 365 nm. Photograph of (**b**) fluorescence from unground metallacycle **91**, (**c**) fluorescence after grinding, and (**d**) after exposure to a dichloromethane vapor. Photograph of (**f**) fluorescence from unground metallacycle **92**, (**g**) fluorescence after grinding, and (**h**) after exposure to a dichloromethane vapor. Reproduced with permission [105]. Copyright 2022 American Chemical Society.

In 2022, Liu, Sun, Wang and co-workers prepared three hexagonal Pt(II) metallacycles **90**–**92** by coordination-driven self-assembly of a 120° di-Pt(II) acceptor **49** with three different 120° dipyridyl donors **87**–**89** bearing TPA, carbazole, or TPE units, respectively (Scheme 16) [105]. The TPA-based metallacycle **90** exhibited AIEE properties. Upon increasing the hexane fraction in CH_2_Cl_2_, its quantum yield increased 90-fold from 0.17% to 15.23%. In the solid state, grinding caused no change in the emission of **90**. In contrast, the carbazole- and TPE-based metallacycles **91** and **92** displayed strong fluorescence both in dilute solution and in aggregates, with quantum yields of 66% and 68% in pure CH_2_Cl_2_, respectively. Both metallacycles showed reversible mechanochromic behavior. As depicted in Figure 12a–d for metallacycle **91**, upon grinding the emission maximum red-shifted from 543 to 554 nm, and the original emission was restored by CH_2_Cl_2_ vapor treatment. A similar red-shift from 530 to 556 nm was observed for metallacycle **92** (Figure 12e–h). Powder X-ray diffraction showed that grinding converted metallacycles **91** and **92** from crystalline to amorphous states, with crystallinity restored by CH_2_Cl_2_ vapor, explaining their mechanochromic fluorescence; metallacycle **90** showed no structural change, consistent with its lack of response.

Overall, the tunable fluorescence of TPA-based metallacycles provides a solid foundation for their functional applications. The ability to modulate emission colors across a broad spectral range, together with high quantum yields in selected systems, makes these metallacycles promising candidates for smart fluorescent materials. The integration of stimuli-responsive behaviors further expands their potential in sensing, imaging, and optoelectronic devices.

### 3.3. Diverse Applications of TPA-Based Metallacycles

The structural diversity, tunable fluorescence, and facile hierarchical assembly of TPA-based metallacycles endow them with significant advantages for application development. These features enable their use in selective sensing, light-harvesting, optoelectronics, and supramolecular theranostics. Collectively, TPA-based metallacycles represent versatile functional materials with promising potential across these diverse frontiers.

**Scheme 17:**
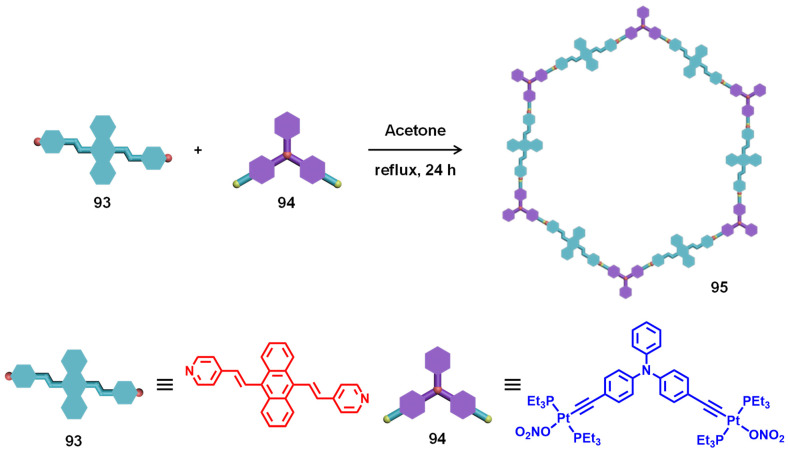
Coordination-driven self-assembly of hexagonal metallacycle **95**.

**Figure 13:**
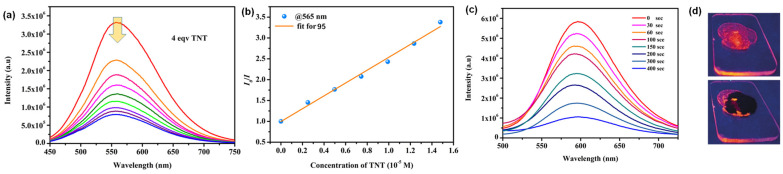
Emission spectra of metallacycle **95** upon addition of increasing amount of TNT (**a**), and SV plot (**b**); *λ*_ex_ = 425 nm. Solid-state quenching of fluorescence intensity of metallacycle **95** with NB vapour (**c**). Fluorescence images of thin films of metallacycle **95** after exposure to NB (**d**). Reproduced with permission [106]. Copyright 2016 John Wiley and Sons.

The detection of nitroaromatic explosives is crucial for public security and environmental protection. The electron-rich TPA and anthracene units can interact with electron-deficient nitroaromatic compounds via PET, leading to fluorescence quenching, which makes them promising building blocks for fluorescent sensors. In 2016, Mukherjee et al. assembled a hexagonal Pt(II) metallacycle **95** from a 120° TPA-based Pt(II) acceptor **94** and a 180° divinylanthracene-bridged dipyridyl donor **93** (Scheme 17) [106]. In CH_2_Cl_2_/hexane mixtures, metallacycle **95** exhibited AIE activity with increasing hexane fraction. Taking advantage of its strong emission and the electron-rich nature of the TPA and anthracene moieties, metallacycle **95** was employed as a fluorescent sensor for nitroaromatic explosives. In solution, its emission was efficiently quenched by trinitrotoluene (TNT) with a Stern–Volmer constant of 1.54 × 10^4^ M^−1^ (Figure 13a,b). Thin films of **95** showed rapid fluorescence quenching upon exposure to saturated vapor of nitrobenzene (NB), and the response was reversible over multiple cycles (Figure 13c,d).

**Scheme 18:**
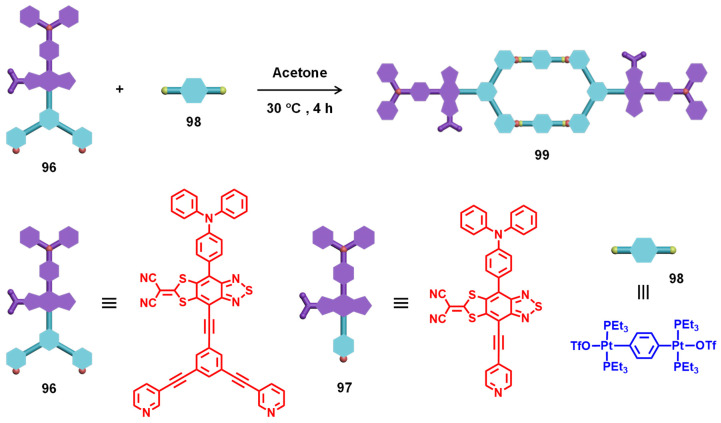
Coordination-driven self-assembly of metallacycles **99**.

**Figure 14:**
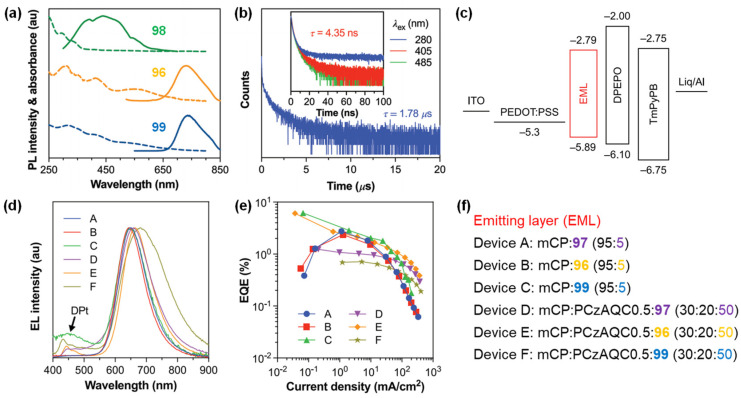
Absorption (dash line) and PL (solid line) spectra of **98**, **96**, and **99** in films, *λ*_ex_ = 500 nm (**a**). PL decay curves of **99** film recorded at 750 nm (**b**). Schematic representation and energy diagram of the OLEDs (**c**). Normalized EL spectra (**d**) and EQE versus current density (**e**) of six devices. Six devices are listed with different emitting layers (**f**). Reproduced with permission [107]. Copyright 2022 John Wiley and Sons.

Two-dimensional metallacycles are an effective supramolecular platform for combining different functional groups to achieve desired functions. In 2021, Tang et al. constructed a discrete Pt(II) metallacycle **99** by self-assembling a 180° di-Pt(II) acceptor **98** with a pyridyl-decorated ligand **96** containing a TPA-based deep-red emitter (Scheme 18) [107]. In the film state, **99** exhibited deep-red emission at 735 nm with a quantum yield of 8%, which is lower than that of the free ligand **96** (740 nm, 24%) due to planarization-induced π–π stacking. Notably, the Pt(II) acceptor **98** served as a triplet sensitizer, enabling efficient intramolecular FRET from the Pt center to the fluorescent emitter (Figure 14a). The distance between the donor and acceptor moieties in **99** was calculated to be about 13.8 Å, which exceeds the typical Dexter transfer range (within 10 Å) and thus favors FRET. Transient PL decay measurements showed a long-lived component of 1.78 μs when exciting the Pt unit, confirming triplet harvesting by the fluorescent emitter (Figure 14b). The electroluminescent devices were fabricated with the structure indium tin oxide (ITO)/poly(3,4-ethylenedioxythiophene):poly(styrene sulfonate) (PEDOT:PSS) (70 nm)/EML (40 nm)/bis [2-(diphenyl phosphino)phenyl] ether oxide (DPEPO) (10 nm)/1,3,5-tri(m-pyridin-3-ylphenyl)benzene (TmPyPB) (50 nm)/8-hydroxyquinolinolato-lithium (Liq)/Al. The emitting layer (EML) consisted of a 1,3-bis(*N*-carbazolyl)benzene (mCP) host blended with either the free ligands (binary system, devices A and B) or the metallacycle (device C), or with additional PCzAQC0.5 sensitizer (ternary system, devices D–F). When metallacycle **99** was used as the emitter in device C, the device achieved a maximum external quantum efficiency (*η*_EQE_) of 6.2% with an emission peak at 650 nm, and an exciton utilization efficiency of 56%, which is more than double the 25% theoretical upper limit for singlet exciton generation in conventional fluorescent emitters (Figure 14c–e). In stark contrast, devices A and B, based on the free ligands (**96** and its mono-pyridyl analogue **97**), gave much lower *η*_EQE_ values (both below 3%). This work demonstrated that TPA-based Pt(II) metallacycles can serve as efficient triplet-harvesting platforms for deep-red/NIR OLEDs.

**Scheme 19:**
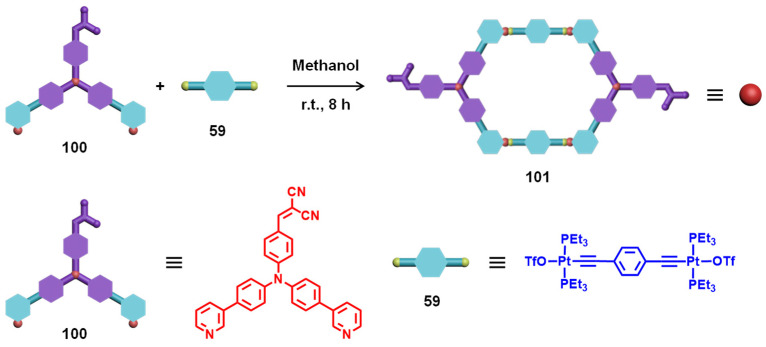
Coordination-driven self-assembly of metallacycle **101**.

**Figure 15:**
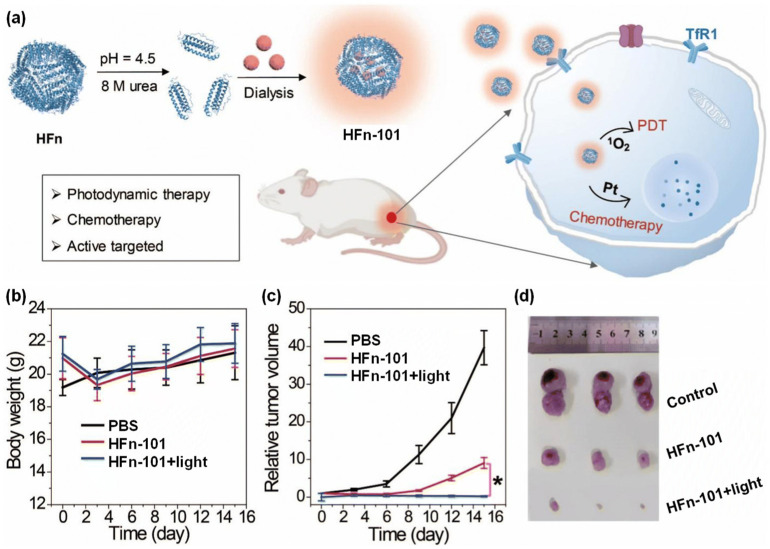
Schematic illustration of the preparation of the HFn-**101** targeted chemo-photodynamic combination therapy after intravenous (i.v.) injection under light irradiation (**a**). The therapeutic effect was studied in CT26 tumor-bearing mice with the HFn-**101**. Mice body weight changes post various treatments (**b**) and tumor growth inhibition curves (**c**), Statistical analysis was performed using a one-way ANOVA followed by Tukey’s multiple comparison test analysis (* *p* < 0.05) to denote significant differences, the mice in the irradiation groups were irradiated with white light (200 mW cm^−2^, 10 min). Photograph of tumors from each group was taken after treatment (**d**). Reproduced with permission [108]. Copyright 2023 Royal Society of Chemistry.

In 2023, Liu, Sun, and co-workers constructed a discrete Pt(II) metallacycle **101** via coordination-driven self-assembly of a 180° di-Pt(II) precursor **59** and a 0° dipyridyl donor **100** bearing a TPA-based dicyanovinyl group (Scheme 19) [108]. The metallacycle **101** combined a platinum-based chemotherapeutic unit and a photosensitizer for reactive oxygen species (ROS) generation. As shown in Figure 15a, to improve water solubility and tumor targeting, hydrophobic **101** was encapsulated into the cavity of human heavy chain ferritin (HFn) through a disassembly–reassembly process, affording HFn-**101** nanogents with a hydrodynamic size of about 13 nm and good stability at physiological pH. HFn-**101** specifically entered transferrin receptor 1 (TfR1)-overexpressing cancer cells (MCF-7, CT26) via receptor-mediated endocytosis, as confirmed by confocal imaging and ICP-MS. Under white light irradiation, HFn-**101** generated singlet oxygen, leading to significant cytotoxicity against CT26 cells, while no obvious toxicity toward normal HFF-1 cells was observed. In CT26 tumor-bearing mice, intravenous injection of HFn-**101** followed by light irradiation effectively suppressed tumor growth without detectable systemic toxicity, as shown by the tumor volume curves and photographs (Figure 15b–d). This work demonstrates that integrating multiple therapeutic functions into a single TPA-based metallacycle offers significant advantages for supramolecular theranostics, enabling precise and synergistic cancer treatment.

**Scheme 20:**
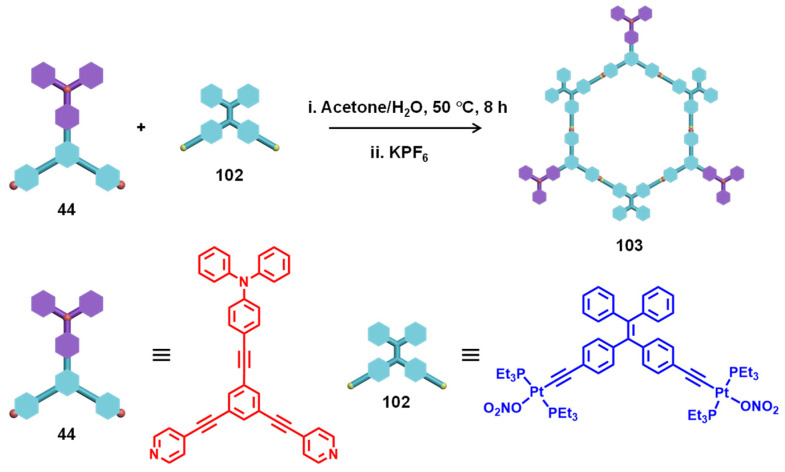
Coordination-driven self-assembly of metallacycle **103**.

**Figure 16:**
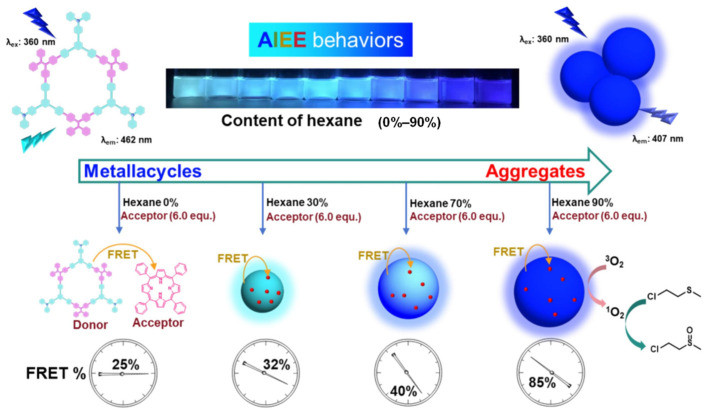
Illustration of tunable FRET signal-based artificial LHS for photocatalytic oxidation. Reproduced with permission [109]. Copyright 2025 Elsevier.

In 2025, Wu, Cheng and co-workers constructed a hexagonal Pt(II) metallacycle **103** via coordination-driven self-assembly of a 120° TPE-containing di-Pt(II) acceptor **102** and a 120° TPA-based dipyridyl donor **44** (Scheme 20) [109]. In CH_2_Cl_2_/hexane mixtures, **103** exhibited AIEE activity as the hexane fraction increased. The emission maximum blue-shifted from 468 nm (0% hexane, cyan) to 407 nm (90% hexane) and the quantum yield increased from 8% to 52% due to restricted intramolecular motion and reduced polarity. The AIEE behavior enabled tuning of FRET from **103** (donor) to tetraphenylporphyrin (**TPP**, acceptor). As the aggregation degree of **103** increased, the spectral overlap and donor–acceptor distance changed, leading to a gradual enhancement of FRET efficiency from 25% (0% hexane) to 85% (90% hexane). As illustrated in Figure 16, the **103–TPP** assembly also served as a photocatalyst for the oxidation of the mustard gas simulant 2-chloroethyl ethyl sulfide (CEES) to its non-toxic sulfoxide. Under light irradiation, the **103–TPP** system achieved 73.8% conversion in 70 min, outperforming **TPP** (67.8%) and **103** alone (20%), demonstrating a synergistic effect. This work provided a strategy to construct tunable artificial LHSs with AIEE-controlled FRET signals for photocatalytic applications.

The application potential of TPA-based metallacycles is derived from their structural tunability. Unlike conventional organic fluorophores or simple coordination complexes, these metallacycles allow for simultaneous modulation of photophysical properties and assembly behaviors within a single well-defined framework through rational molecular design, enabling function-oriented design without compromising structural integrity. This feature, combined with the dynamic nature of coordination bonds, enables the same structural framework to be adapted for diverse application scenarios ranging from chemical sensing to biomedicine.

## 4. Significance of TPA-Based Metallacycles

Within the broad field of SCCs, TPA-based metallacycles have emerged as a distinctive and rapidly developing subclass. They feature structural diversity from three aspects: (i) diverse TPA building blocks with different substituents, functional groups, and linking sites; (ii) diverse metal coordination centers, including Fe(II), Zn(II), Pt(II), and Ru(II), with varied coordination modes; (iii) diverse metallacyclic topologies ranging from rhomboids to hexagons and larger polygons. This structural versatility renders them promising candidates for diverse practical applications. Specifically, the TPA unit serves as an effective directing motif for constructing highly ordered metal–organic assemblies with complex architectures. The favorable optoelectronic properties and synthetic accessibility of TPA derivatives facilitate the incorporation of functional features into the metallacyclic framework. The well-defined 2D topological structures provide unique advantages for hierarchical assembly and function development. Meanwhile, the diversity of metal coordination sites and modes offers an additional avenue for tuning the properties and expanding the application scope of these systems. Collectively, the development of novel functional metallacycles with diverse applications based on the TPA platform remains an important and attractive research topic within the SCCs field.

## 5. Conclusions

In this review, recent advances in TPA-based supramolecular coordination metallacycles have been surveyed, covering five key aspects: coordination-driven self-assembly, hierarchical assembly, supramolecular polymers, tunable fluorescence, and applications. Owing to the rigid structure and facile functionalization of TPA, a variety of metallacycles with well-defined geometries, tunable emissions, and stimuli-responsive behaviors have been constructed. In terms of self-assembly, the approximate 120° angle between the phenyl rings makes TPA a suitable building block for hexagonal and rhomboidal metallacycles. For hierarchical assembly, TPA-based metallacycles can organize into vesicles, nanofibers, and nanospheres driven by π–π stacking, hydrophobic effects, or radical–radical interactions. Regarding supramolecular polymers, the redox activity of TPA enables oxidative electropolymerization to form stable films and porous membranes, while host–guest or dynamic covalent linkages produce fluorescent networks. As for tunable fluorescence, through substituent effects, AIE/AIEE characteristics, and external stimuli (temperature, mechanical force, solvent polarity), these metallacycles exhibit multicolor emission from blue to orange-red with high quantum yields. They have been applied in chemical sensing, light-harvesting, organic light-emitting diodes, and cancer therapy.

Despite the significant progress achieved in this area, several challenges remain to be addressed in future studies. Firstly, the construction of TPA-based metallacycles with more intricate topologies and higher complexity is still highly desirable. Secondly, given the promising anticancer activity of platinum(II) metallacycles and their affinity for biomolecules, TPA-based platinum(II) analogues hold great potential as theranostic platforms that integrate fluorescence imaging and therapy. Thirdly, although TPA is a well-known electron donor widely used in optoelectronic materials (e.g., photothermal agents and TADF emitters), the development of TPA-based metallacycles with such properties remains largely unexplored.

It is clear that TPA-based supramolecular metallacycles will continue to be an active research topic within the SCCs field. Future efforts may focus on exploiting novel structural topologies, enhancing optoelectronic performance, and expanding practical applications across the five highlighted directions. With continued innovation, a bright future for TPA-based supramolecular metallacycles can be anticipated.

## Data Availability

No new data were created or analyzed in this study. Data sharing is not applicable to this article.

## References

[1] Chakrabarty R., Mukherjee P.S., Stang P.J. (2011). Supramolecular coordination: Self-assembly of finite two- and three-dimensional ensembles. Chem. Rev..

[2] Lehn J.M., Rigault A., Siegel J., Harrowfield J., Chevrier B., Moras D. (1987). Spontaneous assembly of double-stranded helicates from oligobipyridine ligands and copper(I) cations: Structure of an inorganic double helix. Proc. Natl. Acad. Sci. USA.

[3] Sauvage J.-P., Dietrich-Buchecker C. (1999). Molecular Catenanes, Rotaxanes, and Knots: A Journey Through the World of Molecular Topology.

[4] Stang P.J., Olenyuk B. (1997). Self-assembly, symmetry, and molecular architecture: Coordination as the motif in the rational design of supramolecular metallacyclic polygons and polyhedra. Acc. Chem. Res..

[5] Fujita M., Tominaga M., Hori A., Therrien B. (2005). Coordination assemblies from a Pd(II)-cornered square complex. Acc. Chem. Res..

[6] Cook T.R., Stang P.J. (2015). Recent developments in the preparation and chemistry of metallacycles and metallacages via coordination. Chem. Rev..

[7] Zarra S., Wood D.M., Roberts D.A., Nitschke J.R. (2015). Molecular containers in complex chemical systems. Chem. Soc. Rev..

[8] Wang W., Wang Y.-X., Yang H.-B. (2016). Supramolecular transformations within discrete coordination-driven supramolecular architectures. Chem. Soc. Rev..

[9] Chakraborty S., Newkome G.R. (2018). Terpyridine-based metallosupramolecular constructs: Tailored monomers to precise 2D-motifs and 3D-metallocages. Chem. Soc. Rev..

[10] Grommet A.B., Feller M., Klajn R. (2020). Chemical reactivity under nanoconfinement. Nat. Nanotechnol..

[11] Sun Y., Chen C., Liu J., Stang P.J. (2020). Recent developments in the construction and applications of platinum-based metallacycles and metallacages via coordination. Chem. Soc. Rev..

[12] Wang H., Li Y., Li N., Filosa A., Li X. (2021). Increasing the size and complexity of discrete 2D metallosupramolecules. Nat. Rev. Mater..

[13] Li X.-Z., Tian C.-B., Sun Q.-F. (2022). Coordination-directed self-assembly of functional polynuclear lanthanide supramolecular architectures. Chem. Rev..

[14] Moree L.K., Faulkner L.A.V., Crowley J.D. (2024). Heterometallic cages: Synthesis and applications. Chem. Soc. Rev..

[15] Yang S.-Y., Chen Y., Kwok R.T.K., Lam J.W.Y., Tang B.Z. (2024). Platinum complexes with aggregation-induced emission. Chem. Soc. Rev..

[16] Tang J.-H., Zhong Y.-W. (2022). Anthracene-containing metallacycles and metallacages: Structures, properties, and applications. Inorganics.

[17] Jia P.-P., Hu Y.-X., Peng Z.-Y., Song B., Zeng Z.-Y., Ling Q.-H., Zhao X., Xu L., Yang H.-B. (2023). Construction of an artificial light-harvesting system with efficient photocatalytic activity in an aqueous solution based on a FRET-featuring metallacage. Inorg. Chem..

[18] Wu G.-Y., Chen L.-J., Xu L., Zhao X.-L., Yang H.-B. (2018). Construction of supramolecular hexagonal metallacycles via coordination-driven self-assembly: Structure, properties and application. Coord. Chem. Rev..

[19] Hu Y.-X., Zhang X., Xu L., Yang H.-B. (2019). Coordination-driven self-assembly of functionalized supramolecular metallacycles: Highlighted research during 2010–2018. Isr. J. Chem..

[20] Li Y., Yu J.-G., Ma L.-L., Li M., An Y.-Y., Han Y.-F. (2021). Strategies for the construction of supramolecular assemblies from poly-NHC ligand precursors. Sci. China Chem..

[21] Garci A., Castor K.J., Fakhoury J., Do J.-L., Di Trani J., Chidchob P., Stein R.S., Mittermaier A.K., Friščić T., Sleiman H. (2017). Efficient and rapid mechanochemical assembly of platinum(II) squares for guanine quadruplex targeting. J. Am. Chem. Soc..

[22] Gao W.-X., Zhang H.-N., Jin G.-X. (2019). Supramolecular catalysis based on discrete heterometallic coordination-driven metallacycles and metallacages. Coord. Chem. Rev..

[23] Schulze M., Kunz V., Frischmann P.D., Würthner F. (2016). A supramolecular ruthenium macrocycle with high catalytic activity for water oxidation that mechanistically mimics photosystem II. Nat. Chem..

[24] Kunz V., Schulze M., Schmidt D., Würthner F. (2017). Trinuclear ruthenium macrocycles: Toward supramolecular water oxidation mcatalysis in pure water. ACS Energy Lett..

[25] Omoto K., Tashiro S., Shionoya M. (2021). Phase-dependent reactivity and host–guest behaviors of a metallo-macrocycle in liquid and solid-state photosensitized oxygenation reactions. J. Am. Chem. Soc..

[26] Hong T., Zhang Z., Sun Y., Tao J.-J., Tang J.-D., Xie C., Wang M., Chen F., Xie S.-S., Li S. (2020). Chiral metallacycles as catalysts for asymmetric conjugate addition of styrylboronic acids to α, β-enones. J. Am. Chem. Soc..

[27] Jia P.-P., Xu L., Hu Y.-X., Li W.-J., Wang X.-Q., Ling Q.-H., Shi X., Yin G.-Q., Li X., Sun H. (2021). Orthogonal self-assembly of a two-step fluorescence-resonance energy transfer system with improved photosensitization efficiency and photooxidation activity. J. Am. Chem. Soc..

[28] Chaudhari K.R., Wadawale A.P., Pathak A.K., Dey S. (2024). Linkage isomers of triangular Pd metallacycles and catalysis in aqueous suzuki coupling reaction. Inorg. Chem..

[29] Wang P., Li S., Liu F.-Z., Yan K.K. (2026). Mechanochemical synthesis of bent metallacycles and confinement catalysis in the solid-state. RSC Mechanochem..

[30] Dou W.-T., Yang H.-B., Xu L. (2025). Fluorescent metallacycles via coordination-driven self-assembly: Preparation, regulation, and applications. Acc. Chem. Res..

[31] Jia P.-P., Hu Y.-X., Lou Z.-C., Ji X., Xu X.-D., Sun H., Jin T., Zheng F., Xu L. (2025). Construction of BODIPY-based triangular metallacycles with tunable photosensitization efficiency. Chin. Chem. Lett..

[32] Frischmann P.D., Mahata K., Würthner F. (2013). Powering the future of molecular artificial photosynthesis with light-harvesting metallosupramolecular dye assemblies. Chem. Soc. Rev..

[33] Huang C.-B., Xu L., Zhu J.-L., Wang Y.-X., Sun B., Li X., Yang H.-B. (2017). Real-time monitoring the dynamics of coordination-driven self-assembly by fluorescence-resonance energy transfer. J. Am. Chem. Soc..

[34] Shi Z.T., Hu Y.-X., Hu Z., Zhang Q., Chen S.-Y., Chen M., Yu J.-J., Yin G.-Q., Sun H., Xu L. (2021). Visible-light-driven rotation of molecular motors in discrete supramolecular metallacycles. J. Am. Chem. Soc..

[35] Garypidou A., Ypsilantis K., Plakatouras J.C., Garoufis A. (2023). Dual-emissive rectangular supramolecular Pt(II)-p-biphenyl with 4,4′-bipyridine derivative metallacycles: Stepwise synthesis and photophysical properties. Molecules.

[36] Yu H., Shi J., Li M., Pan G., Tong H., Xu B., Wang M., Tian W. (2022). Discrete platinum (II) metallacycles with inner-and outermodified 9, 10-Distyrylanthracene: Design, self-assembly, and luminescence properties. Inorg. Chem..

[37] Dey N., Haynes C.J. (2021). Supramolecular coordination complexes as optical biosensors. ChemPlusChem.

[38] Sun Y., Stang P.J. (2021). Metallacycles, metallacages, and their aggregate/optical behavior. Aggregate.

[39] Wei P., Yan X., Huang F. (2015). Supramolecular polymers constructed by orthogonal self-assembly based on host-guest and metal-ligand interactions. Chem. Soc. Rev..

[40] Li B., He T., Fan Y., Yuan X., Qiu H., Yin S. (2019). Recent developments in the construction of metallacycle/metallacage-cored supramolecular polymers via hierarchical self-assembly. Chem. Commun..

[41] Zhu Y., Zheng W., Wang W., Yang H.-B. (2021). When polymerization meets coordination-driven self-assembly: Metallo-supramolecular polymers based on supramolecular coordination complexes. Chem. Soc. Rev..

[42] Zhang C.-W., Ou B., Jiang S.-T., Yin G.-Q., Chen L.-J., Xu L., Li X., Yang H.-B. (2018). Cross-linked AIE supramolecular polymer gels with multiple stimuli-responsive behaviours constructed by hierarchical self-assembly. Polym. Chem..

[43] Zhang Q., Chen F., Shen X., He T., Qiu H., Yin S., Stang P.J. (2021). Self-healing metallacycle-cored supramolecular polymers based on a metal-salen complex constructed by orthogonal metal coordination and host-guest interaction with amino acid sensing. ACS Macro Lett..

[44] Brzechwa-Chodzyńska A., Gołdyn M., Walczak A., Harrowfield J.M., Stefankiewicz A.R. (2021). Hydrogen bonding directed self-assembly of a binuclear Ag(I) metallacycle into a 1D supramolecular polymer. Molecules.

[45] Therrien B. (2020). Thermotropic liquid-crystalline materials based on supramolecular coordination complexes. Inorganics.

[46] Hu Y.-X., Hao X., Xu L., Xie X., Xiong B., Hu Z., Sun H., Yin G.-Q., Li X., Peng H. (2020). Construction of supramolecular liquid-crystalline metallacycles for holographic storage of colored images. J. Am. Chem. Soc..

[47] Hu Y.-X., Hao X., Wang D., Zhang Z.-C., Sun H., Xu X.-D., Xie X., Shi X., Peng H., Yang H.-B. (2024). Light-responsive supramolecular liquid-crystalline metallacycle for orthogonal multimode photopatterning. Angew. Chem. Int. Ed..

[48] Chen L., Chen C., Sun Y., Lu S., Huo H., Tan T., Li A., Li X., Ungar G., Liu F. (2020). Luminescent metallacycle-cored liquid crystals induced by metal coordination. Angew. Chem. Int. Ed..

[49] Tuo W., Xu Y., Fan Y., Li J., Qiu M., Xiong X., Li X., Sun Y. (2021). Biomedical applications of Pt (II) metallacycle/metallacage-based agents: From mono-chemotherapy to versatile imaging contrasts and theranostic platforms. Coord. Chem. Rev..

[50] Sun Y., Ding F., Zhou Z., Li C., Pu M., Xu Y., Zhan Y., Lu X., Li H., Yang G. (2019). Rhomboidal Pt(II) metallacycle-based NIR-II theranostic nanoprobe for tumor diagnosis and image-guided therapy. Proc. Natl. Acad. Sci. USA.

[51] Li Y., Huang F.H., Stang P.J., Yin S.C. (2024). Supramolecular coordination complexes for synergistic cancer therapy. Acc. Chem. Res..

[52] Qin Y., Chen L.-J., Dong F., Jiang S.-T., Yin G.-Q., Li X., Tian Y., Yang H.-B. (2019). Light-controlled generation of singlet oxygen within a discrete dual-stage metallacycle for cancer therapy. J. Am. Chem. Soc..

[53] Yu G.C., Zhang M.M., Saha M.L., Mao Z.W., Chen J., Yao Y., Zhou Z.J., Liu Y.J., Gao C.Y., Huang F.H. (2017). Antitumor activity of a unique polymer that incorporates a fluorescent self-assembled metallacycle. J. Am. Chem. Soc..

[54] Li C., Jia P.-P., Xu Y.-L., Ding F., Yang W.-C., Sun Y., Li X.-P., Yin G.-Q., Xu L., Yang G.-F. (2021). Photoacoustic imaging-guided chemo-photothermal combinational therapy based on emissive Pt (II) metallacycle-loaded biomimic melanin dots. Sci. China Chem..

[55] Zhang J., Yu J., Li W., Fan Y., Li Y., Sun Y., Yin S., Stang P.J. (2022). A near-infrared BODIPY-based rhomboidal metallacycle for imaging-guided photothermal therapy. Inorganics.

[56] Yin C., Du J., Olenyuk B., Stang P.J., Sun Y. (2023). The applications of metallacycles and metallacages. Inorganics.

[57] Hu Y.-X., Li W.-J., Jia P.-P., Wang X.-Q., Xu L., Yang H.-B. (2020). Supramolecular artificial light–harvesting systems with aggregation–induced emission. Adv. Opt. Mater..

[58] Sun Y., Tuo W., Stang P.J. (2022). Metal-organic cycle-based multistage assemblies. Proc. Natl. Acad. Sci. USA.

[59] Tuo W., Sun Y., Lu S., Li X., Sun Y., Stang P.J. (2020). Pillar[5]arene-containing metallacycles and host-guest interaction caused aggregation-induced emission enhancement platforms. J. Am. Chem. Soc..

[60] Hu Y.-X., Jia P.-P., Zhang C.-W., Xu X.-D., Niu Y., Zhao X., Xu Q., Xu L., Yang H.-B. (2021). A supramolecular dual-donor artificial light-harvesting system with efficient visible light-harvesting capacity. Org. Chem. Front..

[61] Hu Y.-X., Wu G.-Y., Wang X.-Q., Yin G.-Q., Zhang C.-W., Li X., Xu L., Yang H.-B. (2021). Acid-activated motion switching of DB24C8 between two discrete Platinum(II) metallacycles. Molecules.

[62] Chen L.-J., Yang H.-B. (2018). Construction of stimuli-responsive functional materials via hierarchical self-assembly involving coordination interactions. Acc. Chem. Res..

[63] Datta S., Saha M.L., Stang P.J. (2018). Hierarchical assemblies of supramolecular coordination complexes. Acc. Chem. Res..

[64] Song Z.-Y., Guo X.-Q., Sun Q.-F. (2026). Hierarchical self-assembly of discrete metal–organic supramolecules with emergent properties and functions. Chem. Rev..

[65] Manifar T., Rohani S. (2004). Synthesis and analysis of triphenylamine: A review. Can. J. Chem. Eng..

[66] He Y.-P., Tan Y.-X., Zhang J. (2020). Functional metal-organic frameworks constructed from triphenylamine-based polycarboxylate ligands. Coord. Chem. Rev..

[67] Chuyko I.A., Troshin P.A., Ponomarenko S.A., Luponosov Y.N. (2025). Polymers based on triphenylamine: Synthesis, properties, and applications. Russ. Chem. Rev..

[68] Bhattacharyya S., Chowdhury A., Saha R., Mukherjee P.S. (2019). Multifunctional self-assembled macrocycles with enhanced emission and reversible photochromic behavior. Inorg. Chem..

[69] Yin Y., Chen Z., Li R.-H., Yuan C., Shao T.-Y., Wang K., Tan H., Sun Y. (2021). Ligand-triggered platinum(II) metallacycle with mechanochromic and vapochromic responses. Inorg. Chem..

[70] Farokhi A., Shahroosvand H., Monache G.D., Pilkington M., Nazeeruddin M.K. (2022). The evolution of triphenylamine hole transport materials for efficient perovskite solar cells. Chem. Soc. Rev..

[71] Mahmood A. (2016). Triphenylamine based dyes for dye sensitized solar cells: A review. Sol. Energy.

[72] Chen S.H., Chen Q., Luo S.H., Cao X.Y., Yang G.X., Zeng X.Q., Wang Z.Y. (2021). Progress in design, synthesis and application of triphenylamine-based fluorescent probes. Chin. J. Org. Chem..

[73] Mao L., Zhou M., Shi X., Yang H.-B. (2021). Triphenylamine (TPA) radical cations and related macrocycles. Chin. Chem. Lett..

[74] Ghosh S., Mukherjee P.S. (2008). Self-assembly of a nanoscopic prism via a new organometallic Pt_3_ acceptor and its fluorescent detection of nitroaromatics. Organometallics.

[75] Ghosh S., Gole B., Bar A.K., Mukherjee P.S. (2009). Self-assembly of molecular prisms via Pt_3_ organometallic acceptors and a Pt_2_ organometallic clip. Organometallics.

[76] Shiga T., Oshiro E., Nakayama N., Mitsumoto K., Newton G.N., Nishikawa H., Oshio H. (2013). Dimerized spin-crossover iron(II) complexes as supramolecular anion capsules. Eur. J. Inorg. Chem..

[77] Roberts D.A., Castilla A.M., Ronson T.K., Nitschke J.R. (2014). Post-assembly modification of kinetically metastable Fe^II^_2_L_3_ triple helicates. J. Am. Chem. Soc..

[78] Adeyemo A.A., Shanmugaraju S., Samanta D., Mukherjee P.S. (2016). Template-free coordination-driven self-assembly of discrete hexanuclear prismatic cages employing half-sandwich octahedral Ru^II^_2_ acceptors and triimidazole donors. Inorg. Chim. Acta.

[79] Sinha N., Tan T.T.Y., Peris E., Hahn F.E. (2017). High-fidelity, narcissistic self-sorting in the synthesis of organometallic assemblies from poly-NHC ligands. Angew. Chem. Int. Ed..

[80] Cui Y., Chen Z.-M., Jiang X.-F., Tong J., Yu S.-Y. (2017). Self-assembly and anion sensing of metal-organic [M_6_L_2_] cages from fluorescent triphenylamine tri-pyrazoles with dipalladium(II, II) corners. Dalton Trans..

[81] Lavendomme R., Ronson T.K., Nitschke J.R. (2019). Metal and organic templates together control the size of covalent macrocycles and cages. J. Am. Chem. Soc..

[82] Zhou Y., Li H., Zhu T., Gao T., Yan P. (2019). A highly luminescent chiral tetrahedral Eu_4_L_4_(L′)_4_ cage: Chirality induction, chirality memory, and circularly polarized luminescence. J. Am. Chem. Soc..

[83] Yao Y., Zhou Y., Zhu T., Gao T., Li H., Yan P. (2020). Eu(III) tetrahedron cage as a luminescent chemosensor for rapidly reversible and turn-on detection of volatile amine/NH_3_. ACS Appl. Mater. Interfaces.

[84] Li Y., Rajasree S.S., Lee G.Y., Yu J., Tang J.-H., Ni R., Li G., Houk K.N., Deria P., Stang P.J. (2021). Anthracene–triphenylamine-based platinum(II) metallacages as synthetic light-harvesting assembly. J. Am. Chem. Soc..

[85] Zhao X., Wang H., Li B., Zheng B., Yang D., Xu W., Li X., Yang X.J., Wu B. (2021). Narcissistic self-sorting in anion-coordination driven assemblies. Chem. Commun..

[86] Davies J.A., Ronson T.K., Nitschke J.R. (2024). Triamine and tetramine edge-length matching drives heteroleptic triangular and tetragonal prism assembly. J. Am. Chem. Soc..

[87] Hwang S.-H., Moorefield C.N., Wang P., Fronczek F.R., Courtney B.H., Newkome G.R. (2006). Design, synthesis and photoelectrochemical properties of hexagonal metallomacrocycles based on triphenylamine:[M6(4,4′-bis(2,2′:6′,2″-terpyridinyl)triphenylamine)_6_(X)_12_]; [M = Fe(II),PF_6_^−^ and Zn(II), BF_4_^−^. Dalton Trans..

[88] Zhang Z., Wang H., Wang X., Li Y., Song B., Bolarinwa O., Reese R.A., Zhang T., Wang X.-Q., Cai J. (2017). Supersnowflakes: Stepwise self-assembly and dynamic exchange of rhombus star-shaped supramolecules. J. Am. Chem. Soc..

[89] Wang L., Song B., Khalife S., Li Y., Ming L.-J., Bai S., Xu Y., Yu H., Wang M., Wang H. (2020). Introducing seven transition metal ions into terpyridine-based supramolecules: Self-assembly and dynamic ligand exchange study. J. Am. Chem. Soc..

[90] Zhang Z., Wang H., Shi J., Wang P., Liu C., Wang M., Li X. (2019). Stepwise self-assembly and dynamic exchange of supramolecular snowflakes. Isr. J. Chem..

[91] Bhattacharyya S., Maity M., Chowdhury A., Saha M.L., Panja S.K., Stang P.J., Mukherjee P.S. (2020). Coordination-assisted reversible photoswitching of spiropyran-based platinum macrocycles. Inorg. Chem..

[92] Fink D., Orth N., Ebel V., Gogesch F.S., Staiger A., Linseis M., Ivanović-Burmazović I., Winter R.F. (2020). Self-assembled redox-active tetraruthenium macrocycles with large intracyclic cavities. Organometallics.

[93] Wang L., Liu R., Gu J., Song B., Wang H., Jiang X., Zhang K., Han X., Hao X.Q., Bai S. (2018). Self-assembly of supramolecular fractals from generation 1 to 5. J. Am. Chem. Soc..

[94] Huo G.-F., Shi X., Tu Q., Hu Y.-X., Wu G.-Y., Yin G.-Q., Li X., Xu L., Ding H.-M., Yang H.-B. (2019). Radical-induced hierarchical self-assembly involving supramolecular coordination complexes in both solution and solid states. J. Am. Chem. Soc..

[95] Acharyya K., Bhattacharyya S., Sepehrpour H., Chakraborty S., Lu S., Shi B., Li X., Mukherjee P.S., Stang P.J. (2019). Self-assembled fluorescent Pt(II) metallacycles as artificial light-harvesting systems. J. Am. Chem. Soc..

[96] Acharyya K., Bhattacharyya S., Lu S., Sun Y., Mukherjee P.S., Stang P.J. (2022). Emissive platinum(II) macrocycles as tunable cascade energy transfer scaffolds. Angew. Chem. Int. Ed..

[97] Xu X.-D., Yao C.-J., Chen L.-J., Yin G.-Q., Zhong Y.-W., Yang H.-B. (2016). Facile construction of structurally defined porous membranes from supramolecular hexakistriphenylamine metallacycles through electropolymerization. Chem.-Eur. J..

[98] Yin G.-Q., Chen L.-J., Wang C.-H., Yang H.-B. (2018). Fabrication of neutral supramolecular polymeric films via post-electropolymerization of discrete metallacycles. Chin. J. Chem..

[99] Zhang M., Yin S., Zhang J., Zhou Z., Saha M.L., Lu C., Stang P.J. (2017). Metallacycle-cored supramolecular assemblies with tunable fluorescence including white-light emission. Proc. Natl. Acad. Sci. USA.

[100] Hou Y., Li S., Zhang Z., Chen L., Zhang M. (2020). A fluorescent platinum(II) metallacycle-cored supramolecular network formed by dynamic covalent bonds and its application in halogen ions and picric acid detection. Polym. Chem..

[101] Tang J.H., Sun Y., Gong Z.L., Li Z.Y., Zhou Z., Wang H., Li X., Saha M.L., Zhong Y.W., Stang P.J. (2018). Temperature-responsive fluorescent organoplatinum (II) metallacycles. J. Am. Chem. Soc..

[102] Chen Z., Tang J.-H., Chen W., Xu Y., Wang H., Zhang Z., Sepehrpour H., Cheng G.-J., Li X., Wang P. (2019). Temperature- and mechanical-force-responsive self-assembled rhomboidal metallacycle. Organometallics.

[103] Zhu J.-L., Xu L., Ren Y.-Y., Zhang Y., Liu X., Yin G.-Q., Sun B., Cao X., Chen Z., Zhao X.-L. (2019). Switchable organoplatinum metallacycles with high quantum yields and tunable fluorescence wavelengths. Nat. Commun..

[104] Wu B., Guo Z., Li G., Zhao J., Liu Y., Wang J., Wang H., Yan X. (2021). Synergistic combination of ACQ and AIE moieties to enhance the emission of hexagonal metallacycles. Chem. Commun..

[105] Yin Y., Chen Z., Li R.H., Yi F., Liang X.C., Cheng S.Q., Wang K., Sun Y., Liu Y. (2022). Highly emissive multipurpose organoplatinum (II) metallacycles with contrasting mechanoresponsive features. Inorg. Chem..

[106] Chowdhury A., Howlader P., Mukherjee P.S. (2016). Aggregation-induced emission of platinum (II) metallacycles and their ability to detect nitroaromatics. Chem.-Eur. J..

[107] Xing H., Yu Y., Liu J., Qin P., Lam J.W.Y., Shi B., Xie G., Tang B.Z. (2022). A discrete platinum(II) metallacycle harvesting triplet excitons for solution-processed deep-red organic light-emitting diodes. Adv. Opt. Mater..

[108] Min X., Li M., Zhang W., Li R.H., Zhang Z., Wang P., Su W., Li F., Sun Y., Liu Y. (2023). Pt(II) metallacycles encapsulated by ferritin enable precise cancer combination chemo-photodynamic therapy. J. Mater. Chem. B.

[109] Wu G.-Y., Liu Q.-J., Hu W.-L., Zhang X., Cheng L. (2025). Aggregation-tuned the Förster resonance energy transfer signal between AIEE-active metallacycle and tetraphenylporphyrin. Tetrahedron Lett..

